# Validation of the Health Assessment Tool (HAT) based on four aging cohorts from the Swedish National study on Aging and Care

**DOI:** 10.1186/s12916-024-03454-4

**Published:** 2024-06-10

**Authors:** Ahmad Abbadi, Emmanouil Kokoroskos, Matthew Stamets, Davide L. Vetrano, Nicola Orsini, Sölve Elmståhl, Cecilia Fagerström, Anders Wimo, Anders Sköldunger, Johan Sanmartin Berglund, Christina B. Olsson, Caroline Wachtler, Laura Fratiglioni, Amaia Calderón-Larrañaga

**Affiliations:** 1 Aging Research Center, Department of Neurobiology, Care Sciences and Society, Karolinska Institutet and Stockholm University, Solna, Sweden; 2 Department of Medical Epidemiology and Biostatistics, Karolinska Institutet, Solna, Sweden; 3 Division of Family Medicine and Primary Care, Department of Neurobiology, Care Sciences, and Society, Karolinska Institutet, Huddinge, Sweden; 4 Husläkarmottagning Täby Centrum, Lideta Mälardalen AB, Täby, Sweden; 5 Department of Global Public Health, Karolinska Institutet, Solna, Sweden; 6 Stockholm Gerontology Research Center, Stockholm, Sweden; 7 Division of Geriatric Medicine, Department of Clinical Sciences in Malmö, Lund University, Malmö, Sweden; 8 Faculty of Health and Life Sciences, Linnaeus University, Kalmar, Sweden; 9 Department of Research, Region Kalmar, Kalmar, Sweden; 10 Division of Neurogeriatrics, Department of Neurobiology, Care Sciences and Society, Karolinska Institutet, Huddinge, Sweden; 11 Department of Health, Blekinge Institute of Technology, Karlskrona, Sweden; 12 Division of Physiotherapy, Department of Neurobiology, Care Sciences and Society, Karolinska Institutet, Huddinge, Sweden; 13 Academic Primary Healthcare Centre, Region Stockholm, Stockholm, Sweden

**Keywords:** Geriatric health assessment, Frailty, Aging cohorts, External validation

## Abstract

**Background:**

As global aging accelerates, routinely assessing the functional status and morbidity burden of older patients becomes paramount. The aim of this study is to assess the validity of the comprehensive clinical and functional Health Assessment Tool (HAT) based on four cohorts of older adults (60 + years) from the Swedish National study on Aging and Care (SNAC) spanning urban, suburban, and rural areas.

**Methods:**

The HAT integrates five health indicators (gait speed, global cognition, number of chronic diseases, and basic and instrumental activities of daily living), providing an individual-level score between 0 and 10. The tool was constructed using nominal response models, first separately for each cohort and then in a harmonized dataset. Outcomes included all-cause mortality over a maximum follow-up of 16 years and unplanned hospital admissions over a maximum of 3 years of follow-up. The predictive capacity was assessed through the area under the curve (AUC) using logistic regressions. For time to death, Cox regressions were performed, and Harrell’s *C*-indices were reported. Results from the four cohorts were pooled using individual participant data meta-analysis and compared with those from the harmonized dataset.

**Results:**

The HAT demonstrated high predictive capacity across all cohorts as well as in the harmonized dataset. In the harmonized dataset, the AUC was 0.84 (95% CI 0.81–0.87) for 1-year mortality, 0.81 (95% CI 0.80–0.83) for 3-year mortality, 0.80 (95% CI 0.79–0.82) for 5-year mortality, 0.69 (95% CI 0.67–0.70) for 1-year unplanned admissions, and 0.69 (95% CI 0.68–0.70) for 3-year unplanned admissions. The Harrell’s *C* for time-to-death throughout 16 years of follow-up was 0.75 (95% CI 0.74–0.75).

**Conclusions:**

The HAT is a highly predictive, clinically intuitive, and externally valid instrument with potential for better addressing older adults’ health needs and optimizing risk stratification at the population level.

**Supplementary Information:**

The online version contains supplementary material available at 10.1186/s12916-024-03454-4.

## Background

The proportion of the world’s population over 60 years will nearly double by 2050 [1]. Although prolonged life expectancy is often accompanied by functional limitations and disability [2], findings from the previous decade indicate that aging processes are modifiable [3]. Recent studies suggest that certain complex interventions can successfully improve the quality of life of older people and prevent or even revert frailty [4, 5].

However, not all people age in the same way or at the same pace. The older population is an extremely heterogeneous group [6]; the older the age group, the greater the variation found in cognitive, physical, and sensory function [7]. Moreover, the speed of deterioration and the degree of overlap in and among these different health indicators tend to vary between and within older individuals over time [1]. There is growing agreement among researchers and clinicians that multiple health indicators are needed to capture the complexity and variability of health status in older adults [3]. The single-disease approach is increasingly being complemented by a functional appraisal to more comprehensively assess the healthcare needs of older people [8, 9]. Such a holistic perspective to older adults’ health is supported by mounting theories and evidence, highlighting the need of shifting from the traditional focus on single diseases and specific time-windows towards multidimensional and longitudinal health trajectories. Thus, validated comprehensive geriatric health assessment tools are needed to optimally identify those older individuals at increased risk of accelerated health decline and intensive care needs [6].

Frailty is one of the most evident manifestations of functional impairment in old age, and several indices have been used to assess frailty despite the lack of consensus on whether it reflects a syndrome or a vulnerability state. Nevertheless, most frailty indices categorize older persons in mutually exclusive frailty states [10, 11], which hinders monitoring subtle changes over time. Moreover, among clinicians working in primary care, frailty is seen as difficult to define, with uncertainty about its value as a medical diagnosis, and not free from a negative connotation [12].

The Health Assessment Tool (HAT) is an instrument for comprehensively assessing the health status of older adults on a continuous scale, developed using data from the Swedish National study on Aging and Care in Kungsholmen (SNAC-K) [13]. In order to assess and visualize age-related variations in health after age 60, Santoni et al. developed this instrument integrating five indicators related to physical and cognitive function, chronic diseases, and disabilities in personal and instrumental activities of daily living, and generated HAT-based geriatric health charts for men and women separately [14, 15]. The HAT is better at predicting adverse health outcomes like unplanned hospitalizations and mortality compared to each of its individual components and to other geriatric health indices [16, 17]. However, the HAT is yet to be tested in other environments in order to externally validate its performance.

The goal of this study was to examine the validity of the HAT based on four aging cohorts from the Swedish National study on Aging and Care (SNAC). To that end, we first aimed to replicate the HAT and its related geriatric health charts in each of the four cohorts, investigate its external validity, and then calculate pooled estimates of its predictive capacity. Additionally, we aimed to examine if nation-wide cut-off points of the HAT could be constructed based on a harmonized dataset including all four cohorts.

## Methods

### Study design, population, and data sources

This study is based on data from the four cohorts of the Swedish National Study on Aging and Care: Kungsholmen (SNAC-K), Blekinge (SNAC-B), Skåne (SNAC-GÅS), and Nordanstig (SNAC-N). SNAC-K was conducted in a highly urban setting, and SNAC-GÅS was carried out in both urban and suburban settings, while SNAC-B and SNAC-N took place in rural and suburban settings. SNAC-K was the development cohort that was used to construct the HAT, and the other three cohorts were used for external validation. The SNAC cohort consists of randomly sampled individuals aged 60 years and older, and the baseline assessments were conducted between 2001 and 2004. Participants completed different questionnaires and underwent thorough examinations by physicians, nurses, and psychologists. Participants are followed up with different frequency: every 6 years for participants aged < 78 and every 3 years for participants aged ≥ 78. The study design has been reported elsewhere [18]. The baseline sample size was of 3363 in SNAC-K, 1402 in SNAC-B, 2931 in SNAC-GÅS, and 766 in SNAC-N [19]. However, following exclusion of participants with missing information on studied health indicators, 3096 participants were included in SNAC-K, 1228 in SNAC-B, 2390 in SNAC-GÅS, and 588 in SNAC-N. In total, 7302 participants were included in the harmonized dataset. All cohorts collected the same variables following similar standardized procedures.

Data from participants were linked to the Swedish National Patient Register (NPR) until 31 December 2016, where information on inpatient and specialist outpatient diagnoses is registered using the International Statistical Classification of Diseases and Related Health Problems (International Classification of Diseases, 10th revision; ICD-10). In the case of SNAC-B, the linkage was only possible with the inpatient register from Blekinge Hospital. Moreover, linkage to the Swedish Cause of Death Register enabled integrating the date of death for each SNAC participant until 31 December 2016.

### The Health Assessment Tool (HAT)

The five health indicators and corresponding health dimensions integrated in the HAT are described below:

1. Physical function was measured by SNAC nurses using gait speed. In SNAC-K and SNAC-N, gait speed was assessed using the 6-m test for those who were able to complete it, and the 2.44-m test for those who were slow or had physical difficulties. In SNAC-GÅS, participants were asked to walk 15 m including a turn. In case the participants were not able to conduct the test due to physical limitations (e.g., on wheelchairs), a score of zero was used. All measures were standardized to meters/second (m/s). SNAC-B did not collect gait speed data at baseline (systematic missing); therefore, predictive mean matching imputation was done using information from all cohorts. Age, sex, education level, mortality, and the other health indicators at baseline were used to match participants from SNAC-B with participants from the other three cohorts. To ensure that the results were not biased or skewed due to this imputation, meta-analysis models were constructed with and without SNAC-B data.
2. Cognitive function was measured by SNAC physicians or nurses through the Mini-Mental State Examination. Values range between 30 (best performance) and 0 (worst performance).
3. Disease burden was measured using the count of chronic diseases, which were defined based on the operationalization by Calderón-Larrañaga et al. [20], and collected by SNAC physicians together with diagnoses from the NPR (or Blekinge Hospital register in the case of SNAC-B). Values ranged between 0 (no chronic diseases) and up to a theoretical maximum of 60 (as described by Calderón-Larrañaga et al.).
4. Mild disability was measured as the number of instrumental activities of daily living (I-ADL) a person was unable to perform independently, i.e., grocery shopping, meal preparation, housekeeping, laundry, managing money, using the telephone, taking medications, and using public transportation. In all cohorts, these activities were assessed by SNAC nurses, except for SNAC-GÅS, where this information was self-fulfilled. Values ranged from 0 to a maximum of 8.
5. Severe disability was measured as the number of personal activities of daily living (P-ADL) a person was unable to perform independently, i.e., bathing, dressing, toileting, continence, transferring, and eating. As for the I-ADL, these activities were assessed by SNAC nurses in all cohorts except for SNAC-GÅS, where this information was self-fulfilled. Values ranged from 0 to a maximum of 6.

### Outcomes

All-cause mortality was operationalized as 1-year mortality (yes/no), 3-year mortality, 5-year mortality, and time to death (up to 16 years of follow-up). Unplanned hospital admissions were extracted from the NPR and were operationalized as 1-year unplanned admission (yes/no) and 3-year unplanned admission. All SNAC cohorts had access to these variables, except for SNAC-B, for which unplanned admissions could not be distinguished from planned ones, and we therefore analyzed all types of admissions together for this cohort.

### Statistical analysis

The HAT was constructed based on the five previously mentioned health indicators using nominal response models (NRm) to choose optimal cut-offs for each indicator against a latent health variable. Subsequently, specific weights for the categories of all indicators were derived by regressing the NRm test characteristic curves (i.e., difficulty and discrimination values) against the health indicators, and final HAT scores were transformed into a continuous scale taking a minimum value of 0 and a maximum value of 10, where higher values indicate better health. The detailed methods behind the construction of the HAT have been published previously [15]. To ensure high internal consistency, the models were rerun using 10 equally split random samples. In total, more than 94,000 models were tested using different cut-off points across the five health indicators. The pre-selection of cut-off points was based on previous literature and the distribution of the variables in the different SNAC cohorts. The STATA code to construct the HAT based on the specific cut-off points for each health indicator can be found in the Additional file 1: Document S1. Additionally, test information functions (TIF) were plotted for each selected model to evaluate how a given combination of indicators (and their corresponding cut-offs) discriminated participants’ health, and at what ranges.

We first aimed to replicate the HAT and assess its pooled predictive capacity across the different SNAC cohorts representing a wide range of socio-demographic areas. To that end, the HAT was constructed using individual-level data separately for each cohort, and its predictive capacity was examined through the area under the curve (AUC) of the receiver operating characteristic (ROC) curve using unadjusted logistic regressions; for time to death, unadjusted Cox regressions were performed and Harrell’s *C*-indices were reported. The results were later meta-analyzed using a two-stage individual participant data meta-analysis (IPD-MA). Fixed effects were applied given the assumption that the IPD-MA would produce an estimate approximating the “true” nation-wide predictive capacity of the HAT. The two-stage individual participant data meta-analysis (IPD-MA) was done with and without SNAC-B for the aforementioned reasons. Moreover, to demonstrate the performance of the HAT without the effect of the development cohort, two-stage IPD-MA was repeated excluding SNAC-K data.

We then used a harmonized dataset including all four cohorts to derive Sweden-wide HAT cut-off points. Harmonization was performed following the recommendations of Fortier et al. [21] and Rolland et al. [22]. We used the SNAC-B imputed gait speed in order to homogenize the data into a common dataset. The other health indicators were collected similarly across cohorts, and measurement units were comparable. Following the harmonization of the data and after checking the consistency of all variables, the reconstruction of the HAT was performed according to the method mentioned previously. Additional cut-off points were used based on the distribution of individual health indicators in the harmonized dataset. In total, more than 31,000 models were tested.

Stratified analyses were done by age (< 78 and ≥ 78 years) and sex for all outcomes and across all SNAC cohorts and the harmonized dataset.

Finally, we modeled the HAT-score percentiles by chronological age, separately for men and women, across all cohorts and in the harmonized dataset using logistic quantile regressions. Given that the HAT scores do not follow a linear trend across chronological age, we used cubic splines with four knots to model these relationships more accurately as smooth and continuous predicted curves, which were plotted in so-called HAT-based geriatric charts. These charts could be used to compare an individual’s health status against a reference population at a given time point, but also to detect individual-level changes in HAT scores over time and, eventually, deviations between expected and observed values [15]. Information on the predicted probabilities for the different outcomes was also incorporated into the charts (only the case of 5-year mortality is shown as an example) using contour plots to facilitate interpretability.

The TRIPOD (Transparent Reporting of a multivariable prediction model for Individual Prognosis Or Diagnosis) checklist was followed in the reporting, which can be found in the Additional file 2.

## Results

### Baseline characteristics

Table 1 summarizes the baseline characteristics of the participants, including the distribution of the outcomes. All cohorts had more female participants than males (average: 58.8% females, 41.2% males). The mean age was highest in SNAC-B (75.9 ± 10.9) and lowest in SNAC-GÅS (71.1 ± 9.7). The overall mean age was 73.4 ± 10.4 years. SNAC-K had the highest proportion of highly educated participants (49.4% with high school education and 33.6% with university or higher education), and SNAC-N had the lowest levels of education (4.9% with university or higher education). Additional file 1: Tables S1 and S2 show the baseline characteristics of the participants stratified by sex and age, respectively. Differences between the sexes were minimal. However, differences became stark when stratifying by age, with a larger number of outcome events among the oldest old, who also showed lower educational levels and a higher proportion of females.

**Table 1:** Baseline characteristics of the study population, by SNAC cohort and in the harmonized dataset

	**SNAC-K** **(*n* = 3096)**		**SNAC-B** **(*n* = 1228)**		**SNAC-GÅS** **(*n* = 2390)**		**SNAC-N** **(*n* = 588)**		**Harmonized dataset** **(*n* = 7302)**	
**Age, mean SD**	74.0	10.9	75.9	10.0	71.1	9.7	74.4	9.7	73.4	10.4
**Age, *n* %**										
< 78	1700	54.9	543	44.2	1610	67.4	304	51.7	4157	56.9
≥ 78	1396	45.1	685	55.8	780	32.6	284	48.3	3145	43.1
**Female, *n* %**	1983	64.1	707	57.6	1292	54.1	311	52.9	4293	58.8
**Education, *n* %**										
Primary school or below	526	17.0	697	56.8	915	38.3	447	76.0	2585	35.4
High school	1529	49.4	396	32.2	1022	42.8	112	19.0	3059	41.9
University of higher	1041	33.6	135	11.0	453	19.0	29	4.9	1658	22.7
**Gait speed (m/s), mean SD**	1.0	0.5	1.0	0.4	1.3	0.3	0.9	0.3	1.1	0.4
**MMSE, mean SD**	27.8	4.3	26.3	4.6	26.9	2.8	28.3	3.0	27.3	3.9
**Chronic diseases, mean SD**	4.0	2.4	2.8	2.1	4.7	2.3	2.1	1.8	3.8	2.4
**I-ADL, mean SD**	0.7	1.8	1.0	1.8	0.5	1.2	0.9	1.9	0.7	1.6
**P-ADL, mean SD**	0.2	0.7	0.2	0.9	0.1	0.5	0.1	0.4	0.1	0.7
**1-year mortality, *n* %**	103	3.3	40	3.3	28	1.2	11	1.9	182	2.5
**3-year mortality, *n*%**	363	11.7	152	12.4	137	5.7	71	12.1	723	9.9
**5-year mortality, *n*%**	612	19.8	277	22.6	287	12.0	121	20.6	1297	17.8
**16-year mortality, *n* %**	1551	50.1	869	70.8	1053	44.1	264	44.9	3737	51.2
**1-year unplanned admissions, *n* %**	486	15.7	420	34.2	360	15.1	99	16.8	1365	18.7
**3-year unplanned admissions, *n* %**	1228	34.2	654	53.3	869	36.4	243	41.3	2824	38.7

*SNAC* Swedish National study on Aging and Care, *K* Kungsholmen, *B* Blekinge, *GÅS* Skåne, *N* Nordanstig, *SD* standard deviation, *MMSE* Mini-Mental State Examination, *I-ADL* instrumental activities of daily living, *P-ADL* personal activities of daily living

### HAT construction

The most frequent health indicator categories for the different HAT scores, and the cut-off points for each SNAC cohort as well as for the harmonized dataset are described in Additional file 1: Tables S3–S8. Even if different cut-off points were obtained across cohorts, increasing HAT scores always showed a hierarchical ordering among the five health indicators, whereby values below 5 indicated mild to severe limitations in ADL, values below 3 indicated severe disability, and values from 5 to 10 indicated a gradual increase in the physical and cognitive functioning and a decrease in the number of chronic diseases. In the harmonized dataset, most participants had a HAT score above or equal to 5 (*n* = 6292, 86.2%). The TIF in each SNAC cohort and the harmonized dataset are shown in Additional file 1: Figure S1. The TIFs show coverage over the whole spectrum of theta (i.e., health), with more information concentrated at positive values of theta (i.e., healthier individuals).

### HAT performance across SNAC cohorts

**Fig. 1:**
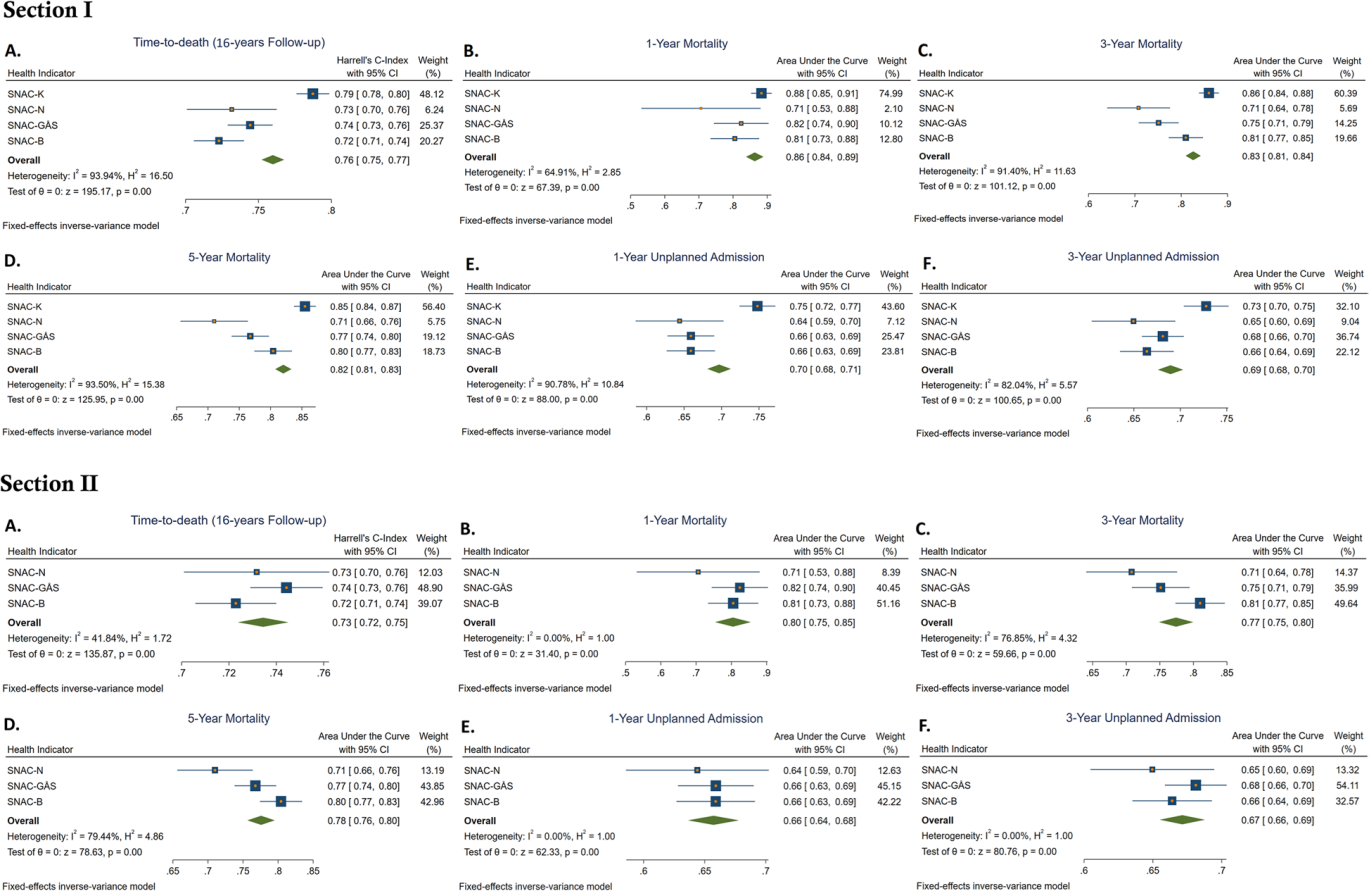
Individual and meta-analyzed predictive capacity of the HAT. Section I across all SNAC cohorts, section II across the external validation cohorts

Figure 1 (section I) shows the predictive capacity of the HAT in each cohort and IPD meta-analyzed for each outcome. Overall, the HAT showed a high predictive capacity in all cohorts, although this was influenced by sample size. The predictive capacity was highest in SNAC-K (i.e., development dataset) but still satisfactory in the external validation datasets. The pooled (i.e., meta-analyzed) Harrell’s *C* for time-to-death was 0.76 (95% CI 0.75, 0.77). The pooled AUC was 0.86 (95% CI 0.84, 0.89) for 1-year mortality, 0.83 (95% CI 0.81, 0.84) for 3-year mortality, and 0.82 (95% CI 0.81, 0.83) for 5-year mortality. The pooled AUC for 1-year unplanned hospital admissions was 0.70 (95% CI 0.68, 0.71), while for 3-year unplanned hospital admissions, it was 0.69 (95% CI 0.68, 0.70). The pooled predictive capacity of the HAT without SNAC-B is shown in Additional file 1: Figure S2. There were no significant differences between the pooled values with or without SNAC-B. Additionally, the pooled predictive capacity of the HAT without SNAC-K is shown in Fig. 1 (section II). With respect to the pooled IPD-MA point estimates for the four cohorts, those without SNAC-K were minimally lower, with differences only in the second decimal of the AUC.

### HAT performance in the harmonized dataset

Table 2 shows the predictive capacity of the HAT for each study outcome in the harmonized dataset, overall and by age and sex. The HAT had high predictive capacity across all outcomes, both in the short and long term. The Harrell’s *C* for time-to-death was 0.75 (95% CI 0.74, 0.75). For 1-year mortality, the AUC was 0.84 (95% CI 0.81, 0.87), while for 3-year mortality, the AUC was 0.81 (95% CI 0.80, 0.83), and for 5-year mortality 0.80 (95% CI 0.79, 0.82). For 1-year unplanned hospital admissions, the AUC was 0.69 (95% CI 0.67, 0.70), as was for 3-year unplanned hospital admissions (0.69, 95% CI 0.68, 0.70). In comparison with the two-stage IPD-MA estimates, the point estimates in the harmonized dataset were slightly lower, but the differences were very small.

**Table 2:** Predictive validity of the HAT in the harmonized dataset, overall and stratified by sex and age

	**Overall**		**Males**		**Females**		** < 78 years**		** ≥ 78 years**	
	**PE**	**(95% CI)**	**PE**	**(95% CI)**	**PE**	**(95% CI)**	**PE**	**(95% CI)**	**PE**	**(95% CI)**
**Time-to-death (16-year follow-up)**^**a**^	0.75	(0.74, 0.75)	0.73	(0.72, 0.74)	0.76	(0.75, 0.77)	0.64	(0.62, 0.66)	0.67	(0.66, 0.68)
**1-year mortality**^**b**^	0.84	(0.81, 0.87)	0.85	(0.81, 0.89)	0.85	(0.81, 0.89)	0.74	(0.60, 0.87)	0.77	(0.73, 0.80)
**3-year mortality**^**b**^	0.81	(0.80, 0.83)	0.80	(0.78, 0.83)	0.83	(0.81, 0.85)	0.69	(0.63, 0.74)	0.74	(0.72, 0.76)
**5-year mortality**^**b**^	0.80	(0.79, 0.82)	0.79	(0.77, 0.81)	0.82	(0.81, 0.84)	0.67	(0.63, 0.71)	0.74	(0.72, 0.75)
**1-year unplanned admission**^**b**^	0.69	(0.67, 0.70)	0.68	(0.65, 0.70)	0.70	(0.68, 0.72)	0.62	(0.59, 0.65)	0.64	(0.62, 0.66)
**3-year unplanned admission**^**b**^	0.69	(0.68, 0.70)	0.68	(0.66, 0.70)	0.70	(0.68, 0.72)	0.61	(0.59, 0.63)	0.63	(0.61, 0.65)

*HAT* Health Assessment Tool, *PE* point estimate, *CI* confidence interval

^a^Harrell’s *C*

^b^Area under the curve (AUC) of the receiver operating characteristic (ROC) curve

Upon sex stratification, the Harrell’s *C* and AUC estimates were slightly higher than the overall estimates among females across all outcomes (e.g., AUC was 0.82 [95% CI 0.81, 0.84] for 5-year mortality among females compared to 0.80 [95% CI 0.79, 0.82] in the overall sample). As for age stratification, those aged ≥ 78 years had higher point estimates compared to those aged < 78 (e.g., for 5-year mortality, those aged ≥ 78 had an AUC of 0.74 while those aged < 78 had an AUC of 0.67). Similar age trends can be seen across SNAC cohorts as shown in Additional file 1: Table S9.

### HAT-based geriatric charts, the example of 5-year mortality

**Fig. 2:**
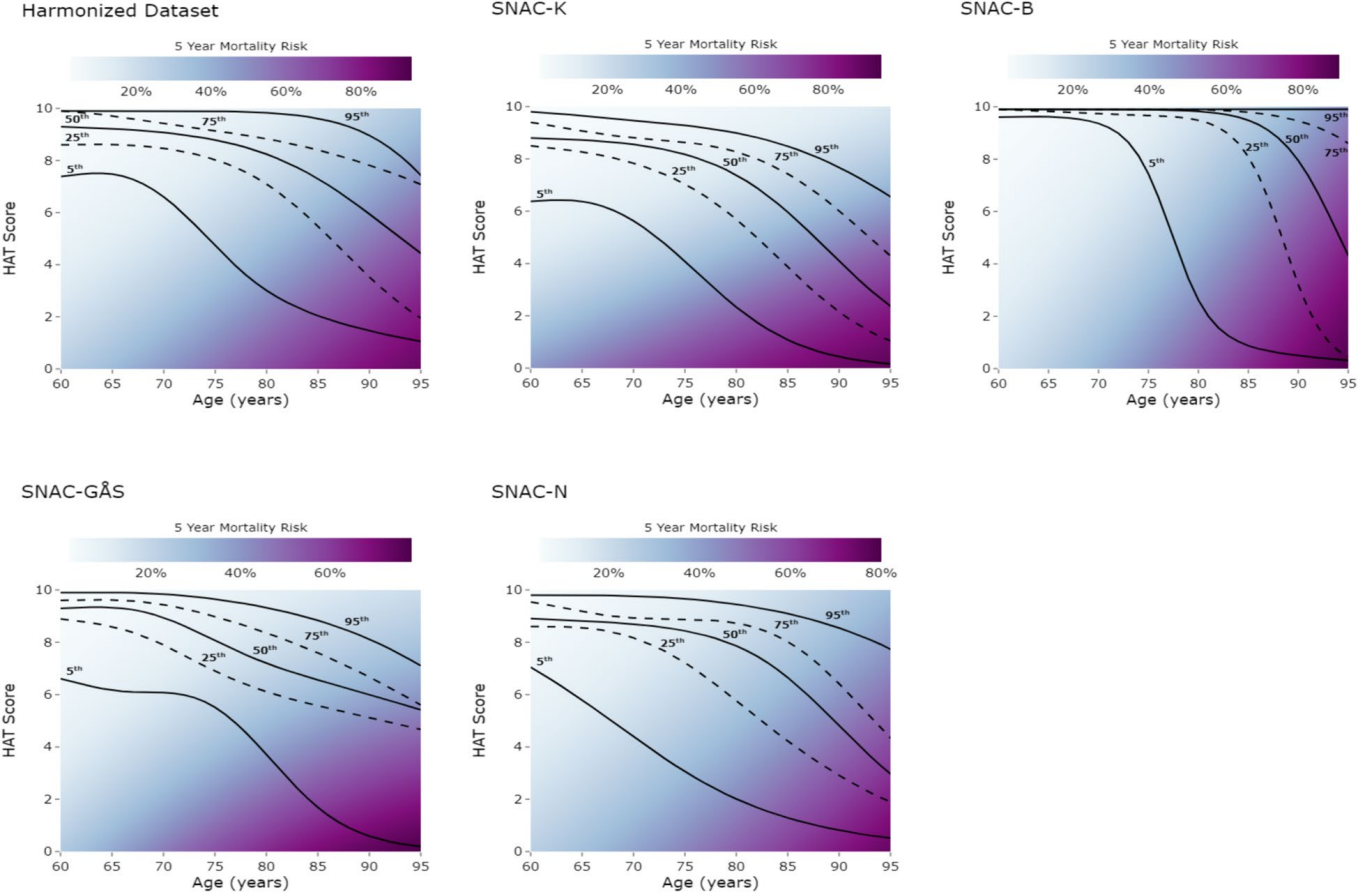
HAT-based geriatric charts and risk of 5-year mortality across all SNAC cohorts and in the harmonized dataset

Figure 2 shows the HAT-based geriatric health charts in each SNAC cohort and in the harmonized dataset, with 5-year mortality as the example outcome. Sex-stratified charts can be found in Additional file 1: Figure S3. The risk of mortality increased significantly with increasing age and decreasing HAT score. For example, at the age of 70 years and for the 95th HAT-score percentile, the risk of 5-year mortality ranged between 4% and 9% across the different SNAC cohorts and was of 6% in the harmonized dataset. However, at the same age of 70 years but for the 5th HAT-score percentile, the risk increased to 8%–21% across cohorts and was of 12% in the harmonized dataset. At the age of 90 years, the 5-year mortality risk ranged between 23% and 37% across cohorts (including the harmonized dataset) for the 95th HAT percentile, it increased to 35%–58% for the 50th HAT percentile, and to 66%–86% for the 5th HAT percentile. Reliability plots for the predictive performance of the models for 5-year mortality are shown in Additional file 1: Figure S4.

## Discussion

The predictive capacity of the HAT was high across all external validation cohorts as well as in the harmonized dataset and was similar in magnitude and direction to that observed in the original development dataset (i.e., SNAC-K), both for mortality as well as unplanned hospital admissions. Even if the predictive capacity was affected by sample size, the method to construct HAT scores was replicable in other settings. Moreover, the performance of the HAT was high in the harmonized dataset, which integrated populations of different age, educational levels, geographic locations, and urbanization levels, similar to the Swedish older population. Furthermore, the HAT-based geriatric health charts are visualization tools that may enable clinicians to view, understand, and communicate HAT scores and their evolution to their patients. The prognosis of different outcomes can be displayed in the charts to guide clinical decisions and treatment plans of patients.

### Comparison with other geriatric health assessment instruments

Few geriatric health assessment instruments have been externally validated, and validation methods vary across studies [23]. Moreover, instruments have usually been developed in inpatient and specialist outpatient clinical environments, using short-term outcomes such as length of hospital stay, 30-day hospital readmissions, or 1-year mortality [24]. Although the HAT was originally aimed at assessing the general health status of older individuals, its components represent key functional domains that overlap with the concept of frailty.

Several tools have been developed to detect frailty, based either on objective or patient-reported indicators, but no consensus exists regarding the tools with best predictive performance [25]. Starting with one of the most well studied instruments, the Frailty Index (FI) by Rockwood and Mitnitski [26], it showed sufficiently good predictive performance in terms of hospitalization and mortality in community-dwelling adults according to the umbrella review by Apóstolo et al. [27], which summarized the existing evidence on the performance of several frailty indices. In a comparative study by Hogan et al. [28], the FI seemed to have modest predictive ability for mortality and hospitalization, with AUC ranging between 0.65 and 0.73 for death and 0.58 and 0.64 for hospitalization in multivariate analyses adjusting for age, sex, and number of chronic conditions. However, according to the findings from the WHO Study on global AGEing and adult health (SAGE) in Shanghai, the AUC of FI for 4-year mortality was over 0.75, but the AUC for 4-year hospitalization was low (AUC 0.53–0.57) [29]. In a study by Vetrano et al. [30], a data-driven Primary Care Frailty Index (PC-FI) including 25 health deficits was developed, which showed a predictive capacity comparable but slightly lower to that of the HAT (*c*-statistic range 0.74–0.84 for mortality and 0.59–0.69 for hospitalization).

Other instruments have also been proposed and tested. The Edmonton Frailty Scale (EFS) has shown good validity and reliability [31], but its use has been limited to the inpatient environment and for disease-specific populations. In a systematic review assessing the construct validity of frailty instruments [32], the EFS and the Clinical Frailty Scale (CFS) were recommended for routine clinical use because of their short administration time and good construct validity. However, no data regarding their predictive ability were given. In a recent multicenter prospective cohort study [33], the authors compared the predictive capacity of the CFS and the Hospital Frailty Risk Score (HFRS) in critically ill patients, and the AUC values for 1-year mortality were 0.66 and 0.63, respectively, and 0.70 for both tools together.

In summary, the predictive capacity of commonly used frailty assessment instruments, whether in the research or clinical settings, is inferior or at best equal to that of the HAT. Unlike in our study, many of the cited papers adjusted for age, sex, and/or other variables, which falsely increases the predictive capacity of the studied tools.

### Clinical and public health implications

The assessment of health status and risk of frailty over time using the HAT could enable clinicians to intervene at the right time and for the right person to slow down the further deterioration of functioning, maintaining capacity or even reversing a declining health trajectory. This can be projected in all four levels of prevention. Older healthy individuals with high HAT scores could get primary preventative advice and be monitored for risk factors for frailty, both at the primary care level as well as through higher level public-health policy. Ki et al. [34] developed a framework for preventing frailty that comprised different domains among which are physical activity, resilience, and management of chronic diseases. In terms of secondary prevention, patients with prefrailty or with reversible frailty could be identified. In recent systematic and scoping reviews, it has been shown that complex primary care interventions, including physical and nutritional counseling and comprehensive geriatric assessment, may effectively reverse frailty and postpone the transition to frailty in prefrail individuals [4, 5]. Unfortunately, though, frailty can also be irreversible. This group of patients with irreversible frailty can get help to reduce the incidence of complications such as disability and dementia and to maintain the best possible quality of life [5], along the spectrum of tertiary and quaternary prevention. Consequently, a geriatric health assessment tool, such as the HAT, which can accurately predict short- and long-term risk of negative outcomes may be of great help for primary care units to better target the level and intensity of prevention and care to their older patients.

Many diagnostic, preventive, and/or therapeutic decisions taken in primary care are done based on patients’ chronological age. In lieu, the HAT has potential to support clinical decision-making based on biological age. For example, screening for colorectal cancer is not recommended for individuals over the age of 75 [35], and PSA testing for cancer is currently not motivated for men over the age of 70 [36]. Primary prevention medications such as statin treatment for modification of the risk for coronary artery disease or other vascular diseases have been questioned after the age of 75 in recent studies [37]. However, such decisions should be individualized, rather than age restricted, based on accurate geriatric health assessments as claimed for in recent initiatives against ageism. Authors of a recent systematic review [38] showed that ageism led to significantly worse health outcomes in 95.5% of the studies included in the review. Strategies to reduce ageistic approaches to treating older patients are warranted.

### Strengths and limitations

The HAT was externally validated in Swedish aging cohorts representing different socio-demographic and urbanization levels. The results of our study have been additionally interpreted after age- and sex-stratified analyses and considering both short- and long-term outcomes. Beyond the individual cohorts, using a harmonized dataset increased the sample size enabling an optimal performance of the statistical models and recalibration of the HAT. The visualization tool proposed may strengthen the clinical applicability of the HAT.

Nevertheless, some limitations need to be acknowledged. Within the SNAC-B cohort, there were two major discrepancies in the measurement of the individual health indicators. First, gait speed was not measured at baseline, which required imputing the values, and second, it was not possible to differentiate between unplanned and planned hospital admissions. However, to alleviate the impact of such limitations, the IPD-MA was done with and without SNAC-B. The results showed that the statistical measures taken did not have any negative effects on the overall results, and that SNAC-B performed similarly as expected based on sample size and distribution. Inconsistencies due to measurement heterogeneity across cohorts were documented. However, such discrepancies reflect the real-world clinical environment and are expected to have minimal impact following standardization and harmonization.

When stratifying by age, the predictive capacity of the HAT decreased, more so among the younger group. Higher functional resilience among the younger-old group could limit the ability to capture poor outcomes by the different components of the HAT [16]. This is evidenced by differences in the distribution of outcome events among the age groups. Complementarily, although the TIF showed high performance across the whole spectrum of the latent health construct, the information density was lower for levels of poorer health. Given that the manifestation of poor health differs among the older and younger groups [14], this might affect the performance of the HAT and could explain the lower predictive capacity when stratifying by age. Additional research is warranted to understand why these differences are observed and their impact on the tools’ predictive capacity.

Lastly, even if the external validity of the HAT was overall very good across different Swedish aging cohorts, it still needs to be tested further in routine clinical (and ideally primary care) practice, both in terms of its predictive capacity as well as its feasibility and acceptability among healthcare professionals and patients.

## Conclusions

The HAT showed a high predictive capacity across different Swedish aging cohorts and outcomes, which corroborates the external validity of the tool. The tool, together with the HAT-based geriatric charts, may assist primary care professionals in decision-making by avoiding ageist behaviors and by facilitating truly patient-centered care for older patients. The HAT may also be used to target frailty prevention and/or reversion interventions to specific groups of patients in primary care, where most older subjects are continuously and comprehensively monitored. Further, the HAT may facilitate better planning of home assistance and social support. Future studies are to evaluate the implementation of the HAT in primary care routine practice and other settings in Sweden and beyond.

### Supplementary Information

Additional file 1: Document S1, Tables S1–S9, Figures S1–S4. Document S1 Calculation of HAT scores, Table S1 Baseline characteristics of the study population across SNAC sites stratified by sex, Table S2 Baseline characteristics of the study population across SNAC sites stratified by age, Table S3 Individual health indicator cut-off points, Table S4 Most frequent health indicator categories by HAT score in SNAC-K, Table S5 Most frequent health indicator categories by HAT score in SNAC-B, Table S6 Most frequent health indicator categories by HAT score in SNAC-GÅS, Table S7 Most frequent health indicator categories by HAT score in SNAC-N, Table S8 Most frequent health indicator categories by HAT score in harmonized dataset, Figure S1 Test information function, Figure S2 Individual and meta-analyzed predictive capacity of the HAT across all SNAC sites except for SNAC-B, Table S9 Predictive capacity of the HAT, Figure S3 HAT-based geriatric charts and risk of 5-year mortality, Figure S4 Reliability plots.

Additional file 2: TRIPOD checklist.

## Data Availability

The datasets generated and/or analyzed in the current study are not publicly available due to ethical and data sharing restrictions/laws, including but not limited to GDPR. The data, however, can be requested formally from the principal investigators of each SNAC cohort.

## Abbreviations

AUC: Area under the curve
CFS: Clinical Frailty Scale
EFS: Edmonton Frailty Scale
FI: Frailty Index
HAT: Health Assessment Tool
HFRS: Hospital Frailty Risk Score
I-ADL: Instrumental activities of daily living
ICD-10: International Classification of Diseases, 10th revision
IPD-MA: Individual participant data meta-analysis
m/s: Meters/second
NPR: National Patient Register
NRm: Nominal response models
P-ADL: Personal activities of daily living
PC-FI: Primary Care Frailty Index
ROC: Receiver operating characteristic
SAGE: Study on global AGEing and adult health
SNAC: Swedish National study on Aging and Care
SNAC-B: Swedish National study on Aging and Care in Blekinge
SNAC-GÅS: Swedish National study on Aging and Care in Skåne
SNAC-K: Swedish National study on Aging and Care in Kungsholmen
SNAC-N: Swedish National study on Aging and Care in Nordanstig
TIF: Test information functions
TRIPOD: Transparent Reporting of a multivariable prediction model for Individual Prognosis Or Diagnosis
