# Supplementary material for: Validation of the Health Assessment Tool (HAT) based on four aging cohorts from the Swedish National study on Aging and Care
Source: BMC Med. 2024 Jun 10;22:236. doi: 10.1186/s12916-024-03454-4 (PMC11165739; doi:10.1186/s12916-024-03454-4)

**Validation of the Health Assessment Tool (HAT) based on four aging cohorts from the Swedish National study on Aging and Care**

**Supplementary Document 1**

Table of Contents

[**Document S1: Calculation of HAT scores across SNAC sites and in the harmonized dataset** 2](#_Toc167381941)

[**Table S1: Baseline characteristics of the study population, by SNAC cohort and in the harmonized dataset stratified by sex.** 4](#_Toc167381942)

[**Table S2: Baseline characteristics of the study population, by SNAC cohort and in the harmonized dataset stratified by age.** 6](#_Toc167381943)

[**Table S3: Individual health indicator cut-off points across all SNAC sites and in the harmonized dataset.** 8](#_Toc167381944)

[**Table S4: Most frequent health indicator categories (frequency 50% and above) by HAT score in SNAC-K.** 9](#_Toc167381945)

[**Table S5: Most frequent health indicator categories (frequency 50% and above) by HAT score in SNAC-B.** 9](#_Toc167381946)

[**Table S6: Most frequent health indicator categories (frequency 50% and above) by HAT score in SNAC-GÅS.** 9](#_Toc167381947)

[**Table S7: Most frequent health indicator categories (frequency 50% and above) by HAT score in SNAC-N.** 10](#_Toc167381948)

[**Table S8: Most frequent health indicator categories (frequency 50% and above) by HAT score in the harmonized dataset.** 10](#_Toc167381949)

[**Figure S1: Test Information Function across all SNAC sites and in the harmonized dataset.** 11](#_Toc167381950)

[**Figure S2: Individual and meta-analyzed predictive capacity of the HAT across all SNAC sites except for SNAC-B.** 13](#_Toc167381951)

[**Table S9: Predictive capacity of the HAT across SNAC sites and in the harmonized dataset, overall and stratified by sex and age.** 14](#_Toc167381952)

[**Figure S3: HAT-based geriatric charts and risk of 5-years mortality stratified by sex across all SNAC sites and in the harmonized dataset.** 17](#_Toc167381953)

[**Figure S4: Reliability plots for different SNAC sites and the harmonized dataset, divided into 10 equal sized groups (percentiles) with 5-year mortality as the outcome.** 20](#_Toc167381954)

# **Document S1: Calculation of HAT scores across SNAC sites and in the harmonized dataset**

To compute HAT score, the following variables are required: personal activities of daily living (P-ADL, also referred to as ADL), instrumental activities of daily living (I-ADL, also referred to as IADL), gait speed expressed in m/s (WW), mini-mental state examination score (MMSE, also referred to as MM), and count of chronic diseases (MCD). The following STATA commands are written using the outputs of linear regression of latent health status (dependent) and HAT score components (independent variables). The latent health status is generated from item response theory nominal response model. The linear regression considered interaction between variables if the power was sufficient. The code uses the constant generated from the linear regression as the base that the subtractions will occur with each deterioration in HAT component. The value of each HAT component was determined based on the cut-off points listed in Table 2 and Supplementary Table 1. The categories start from 0 and go up with worse performance according to the cut-off point.

1. SNAC-K:

forv x=0/0{

gen int t_`x’ = (adl`x'b==0)*(iadl`x'b == 0)*(100+(-7*(MM`x'==1)-13*(MM`x'==2)-8*(WW`x'==1)-27*(WW`x'==2)-32*(WW`x'==3)+1*(MM`x'==1)*(WW`x'==1)+2*(MM`x'==1)*(WW`x'==2)+3*(MM`x'==1)*(WW`x'==3)-9*(MM`x'==2)*(WW`x'==1)-2*(MCD`x'==1)-6*(MCD`x'==2)-11*(MCD`x'==3)+1*(MCD`x'==1)*(WW`x'==1)-5*(MCD`x'==1)*(WW`x'==3)+1*(MCD`x'==2)*(WW`x'==1)+2*(MCD`x'==2)*(WW`x'==2)-2*(MCD`x'==2)*(WW`x'==3)+1*(MCD`x'==3)*(WW`x'==1)+4*(MCD`x'==3)*(WW`x'==2)+1*(MM`x'==1)*(MCD`x'==2)+1*(MM`x'==1)*(MCD`x'==3)))+(adl`x'b==0)*(iadl`x'b == 1)*(61+(-3*(MM`x'==1)-16*(MM`x'==2)-4*(WW`x'==1)-15*(WW`x'==2)-25*(WW`x'==3)+2*(MM`x'==2)*(WW`x'==2)-2*(MCD`x'==2)-5*(MCD`x'==3)+1*(MM`x'==2)*(MCD`x'==2)))+(adl`x'b==1)*(iadl`x'b == 0)*(77+(-4*(MM`x'==1)-14*(MM`x'==2)-6*(WW`x'==1)-18*(WW`x'==2)-27*(WW`x'==3)+1*(MM`x'==1)*(WW`x'==2)-5*(MCD`x'==3)+1*(MCD`x'==3)*(WW`x'==1)+2*(MCD`x'==3)*(WW`x'==2)+1*(MCD`x'==3)*(WW`x'==3)))+(adl`x'b==1)*(iadl`x'b == 1)*(33+(-3*(MM`x'==1)-16*(MM`x'==2)-10*(WW`x'==3)-1*(MM`x'==1)*(WW`x'==3)-3*(MM`x'==2)*(WW`x'==3)-3*(MCD`x'==3)))

gen double Sc`x' = t_`x'/10

}

1. SNAC-B

forv x=0/0 {

gen int t_`x' = (adl`x'b==0)*(iadl`x'b==0) *(99-2*(MM`x'==1)*(WW`x'==3)-1*(MM`x'==2)*(WW`x'==2)-6*(MM`x'==2)*(WW`x'==3)-1*(MCD`x'==1)*(WW`x'==3)-1*(MCD`x'==1)*(MM`x'==2))+((adl`x'b>0)*(iadl`x'b==0))*(96-13*(WW`x'==3)-15*(MM`x'==2)-3*(MCD`x'==1)) + ((adl`x'b==0)*(iadl`x'b>0))*(74-4*(WW`x'==2)-19*(WW`x'==3)-7*(MM`x'==1)-19*(MM`x'==2)-4*(MCD`x'==1)) + ((adl`x'b>0)*(iadl`x'b>0))*(44-17*(WW`x'==3)-7*(MM`x'==1)-22*(MM`x'==2)-5*(MCD`x'==1))

gen double Sc`x' = t_`x'/10

}

1. SNAC-GÅS

forv x=0/0 {

gen int t_`x' = (adl`x'b==0) * (100-2*(MM`x'==2)-9*(MM`x'==3)-1*(WW`x'==1)-9*(WW`x'==2)-24*(WW`x'==3)-1*(MM`x'==2)*(WW`x'==1)-3*(MM`x'==2)*(WW`x'==2)-3*(MM`x'==2)*(WW`x'==3)-3*(MM`x'==3)*(WW`x'==1)-7*(MM`x'==3)*(WW`x'==2)-5*(MM`x'==3)*(WW`x'==3)-1*(MCD`x'==1)-4*(MCD`x'==2)-1*(MCD`x'==1)*(WW`x'==1)-3*(MCD`x'==1)*(WW`x'==2)-1*(MCD`x'==1)*(WW`x'==3)-2*(MCD`x'==2)*(WW`x'==1)-6*(MCD`x'==2)*(WW`x'==2)-6*(MCD`x'==2)*(WW`x'==3)-1*(MCD`x'==1)*(MM`x'==2)+1*(MCD`x'==1)*(MM`x'==3)-1*(MCD`x'==2)*(MM`x'==2)-2*(iadl`x'b==1)-25*(iadl`x'b==2)+1*(iadl`x'b==2)*(MM`x'==1)+2*(iadl`x'b==2)*(MM`x'==2)+5*(iadl`x'b==2)*(MM`x'==3)-1*(iadl`x'b==1)*(MCD`x'==1)-1*(iadl`x'b==1)*(MCD`x'==2)-2*(iadl`x'b==2)*(MCD`x'==1)-1*(iadl`x'b==1)*(WW`x'==1)-2*(iadl`x'b==1)*(WW`x'==2)-2*(iadl`x'b==1)*(WW`x'==3)+1*(iadl`x'b==2)*(WW`x'==1)+3*(iadl`x'b==2)*(WW`x'==3)) +((adl`x'b>0))*(20-1*(MM`x'==2)-4*(MM`x'==3)-4*(WW`x'==3)-1*(MCD`x'==1)-3*(MCD`x'==2)-2*(iadl`x'b==1)-8*(iadl`x'b==2))

gen double Sc`x' = t_`x'/10

}

1. SNAC-N

forv x=0/0{

gen int t_`x' = (iadl`x'b==0)*(99-4*(MM`x'==1)-11*(MM`x'==2)-17*(MM`x'==3)-11*(WW`x'==1)-29*(WW`x'==2)-4*(MCD`x'==1)-8*(MCD`x'==2)-19*(adl`x'b==1)) + (iadl`x'b>0)*(68-3*(MM`x'==1)-9*(MM`x'==2)-15*(MM`x'==3)-9*(WW`x'==1)-26*(WW`x'==2)-3*(MCD`x'==1)-7*(MCD`x'==2)-18*(adl`x'b==1))

gen double Sc`x' = t_`x'/10

}

1. Harmonized dataset

forv x=0/0 {

gen int t_`x' = (adl`x'b==0)*(iadl`x'b==0)*(100-6*(MM`x'==1)-1*(WW`x'==1)-13*(WW`x'==2)-1*(MCD`x'==1)-7*(MCD`x'==2))+ ((adl`x'b==0)*(iadl`x'b==1))*(89-6*(MM`x'==1)-1*(WW`x'==1)-12*(WW`x'==2)+2*(MM`x'==1)*(WW`x'==2)-2*(MCD`x'==1)-7*(MCD`x'==2)+2*(MCD`x'==2)*(WW`x'==2)+1*(MCD`x'==2)*(MM`x'==1)) + ((adl`x'b==0)*(iadl`x'b==2))*(47-4*(MM`x'==1)+1*(WW`x'==1)-8*(WW`x'==2)-1*(MM`x'==1)*(WW`x'==1)-5*(MCD`x'==2)-1*(MCD`x'==1)*(WW`x'==1)-1*(MCD`x'==1)*(WW`x'==2)-1*(MCD`x'==2)*(WW`x'==1)) + ((adl`x'b==1)*(iadl`x'b==0))*(78-4*(MM`x'==1)-8*(WW`x'==2)-1*(MM`x'==1)*(WW`x'==1)-1*(MCD`x'==1)-4*(MCD`x'==2)-1*(MCD`x'==1)*(WW`x'==1)-1*(MCD`x'==2)*(WW`x'==1)+1*(MCD`x'==1)*(MM`x'==1)) + ((adl`x'b==1)*(iadl`x'b==1))*(71-4*(MM`x'==1)-8*(WW`x'==2)-1*(MCD`x'==1)-5*(MCD`x'==2)) + ((adl`x'b==1)*(iadl`x'b==2))*(34-4*(MM`x'==1)-7*(WW`x'==2)-1*(MCD`x'==1)-4*(MCD`x'==2)) + ((adl`x'b==2)*(iadl`x'b<2))*(44) + ((adl`x'b==2)*(iadl`x'b==2))*(20-5*(MM`x'==1)-9*(WW`x'==2)-1*(MCD`x'==1)-6*(MCD`x'==2))

gen double Sc`x' = t_`x'/10

}

# **Table S1: Baseline characteristics of the study population, by SNAC cohort and in the harmonized dataset stratified by sex.**

| **Males** |  | | | | | | | | | |  |
| --- | --- | --- | --- | --- | --- | --- | --- | --- | --- | --- | --- |
|  | **SNAC-K**  **(n=1,113)** | | **SNAC-B**  **(n=521)** | | **SNAC-GÅS**  **(n=1,098)** | | **SNAC-N**  **(n=278)** | | **Harmonized dataset (n=3,009)** | |  |
| **Age, mean SD** | 71.5 | 10.0 | 74.9 | 9.9 | 70.1 | 9.4 | 73.6 | 9.6 | 71.8 | 9.9 |  |
| **Education, n %** |  |  |  |  |  |  |  |  |  |  |  |
| Primary school or below | 146 | 13.1 | 261 | 50.1 | 423 | 38.5 | 211 | 75.9 | 1,040 | 34.6 |  |
| High school | 470 | 42.2 | 191 | 36.7 | 440 | 40.1 | 56 | 20.1 | 1,157 | 38.5 |  |
| University of higher | 497 | 44.7 | 69 | 13.2 | 235 | 21.4 | 11 | 4.0 | 812 | 27.0 |  |
| **Gait speed (m/s), mean SD** | 1.1 | 0.4 | 1.1 | 0.4 | 1.3 | 0.3 | 0.9 | 0.3 | 1.2 | 0.4 |  |
| **MMSE, mean SD** | 28.4 | 3.2 | 26.8 | 3.8 | 26.9 | 2.8 | 28.2 | 2.8 | 27.5 | 3.2 |  |
| **Chronic diseases, mean SD** | 3.6 | 2.4 | 2.4 | 1.9 | 4.4 | 2.1 | 2.0 | 1.7 | 3.5 | 2.3 |  |
| **I-ADL, mean SD** | 0.5 | 1.4 | 1.0 | 1.7 | 0.6 | 1.2 | 0.9 | 2.0 | 0.6 | 1.5 |  |
| **P-ADL, mean SD** | 0.1 | 0.5 | 0.1 | 0.7 | 0.1 | 0.4 | 0.1 | 0.3 | 0.1 | 0.5 |  |
| **1-year mortality, n %** | 35 | 3.1 | 25 | 4.8 | 18 | 1.6 | 7 | 2.5 | 85 | 2.8 |  |
| **3-year mortality, n%** | 119 | 10.7 | 71 | 13.6 | 74 | 6.7 | 35 | 12.5 | 299 | 9.9 |  |
| **5-year mortality, n%** | 209 | 18.8 | 124 | 23.8 | 162 | 14.8 | 60 | 21.5 | 555 | 18.4 |  |
| **16-year mortality, n %** | 538 | 48.3 | 326 | 62.6 | 507 | 46.2 | 127 | 45.5 | 1,545 | 51.4 |  |
| **1-year unplanned admissions, n %** | 176 | 15.8 | 164 | 31.5 | 192 | 17.5 | 52 | 18.6 | 584 | 19.4 |  |
| **3-year unplanned admissions, n %** | 369 | 33.2 | 264 | 50.7 | 416 | 37.9 | 122 | 43.7 | 1,170 | 38.9 |  |
| **Females** | |  | | | | | | | | | |
|  | | **SNAC-K**  **(n=1,983)** | | **SNAC-B**  **(n=707)** | | **SNAC-GÅS**  **(n=1,292)** | | **SNAC-N**  **(n=311)** | | **Harmonized dataset (n=4,293)** | |
| **Age, mean SD** | | 75.4 | 11.2 | 76.6 | 10.1 | 71.9 | 9.9 | 75.1 | 9.7 | 74.5 | 10.7 |
| **Education, n %** | |  |  |  |  |  |  |  |  |  |  |
| Primary school or below | | 380 | 19.1 | 436 | 61.7 | 492 | 38.1 | 237 | 76.2 | 1,545 | 36.0 |
| High school | | 1,059 | 53.4 | 205 | 29.0 | 582 | 45.1 | 56 | 18.0 | 1,902 | 44.3 |
| University of higher | | 544 | 27.43 | 66 | 9.3 | 218 | 16.9 | 18 | 5.8 | 846 | 19.7 |
| **Gait speed (m/s), mean SD** | | 0.9 | 0.5 | 1.0 | 0.4 | 1.3 | 0.3 | 0.9 | 0.3 | 1.0 | 0.4 |
| **MMSE, mean SD** | | 27.5 | 4.8 | 25.9 | 5.0 | 26.9 | 2.9 | 28.3 | 3.1 | 27.1 | 4.3 |
| **Chronic diseases, mean SD** | | 4.2 | 2.5 | 3.1 | 2.3 | 4.9 | 2.4 | 2.1 | 1.8 | 4.1 | 2.5 |
| **I-ADL, mean SD** | | 0.9 | 1.9 | 1.1 | 1.9 | 0.4 | 1.1 | 0.9 | 1.9 | 0.8 | 1.7 |
| **P-ADL, mean SD** | | 0.2 | 0.8 | 0.3 | 1.0 | 0.1 | 0.5 | 0.1 | 0.4 | 0.2 | 0.7 |
| **1-year mortality, n %** | | 68 | 3.4 | 15 | 2.1 | 10 | 0.8 | 4 | 1.3 | 97 | 2.3 |
| **3-year mortality, n%** | | 244 | 12.3 | 81 | 11.5 | 63 | 4.9 | 36 | 11.6 | 424 | 9.9 |
| **5-year mortality, n%** | | 403 | 20.3 | 153 | 21.6 | 125 | 9.7 | 61 | 19.6 | 742 | 17.3 |
| **16-year mortality, n %** | | 1,013 | 51.1 | 419 | 59.3 | 546 | 42.3 | 138 | 44.4 | 2,192 | 51.1 |
| **1-year unplanned admissions, n %** | | 310 | 15.6 | 420 | 34.2 | 168 | 13.0 | 47 | 15.1 | 781 | 18.2 |
| **3-year unplanned admissions, n %** | | 689 | 34.8 | 654 | 53.3 | 453 | 35.1 | 122 | 39.2 | 1,654 | 38.5 |
| SNAC: Swedish National study on Aging and Care, K: Kungsholmen, B: Blekinge, GÅS: Skåne, N: Nordanstig, SD: standard deviation, MMSE: Mini-mental state examination, I-ADL: instrumental activities of daily living, P-ADL: Personal activities of daily living. | | | | | | | | | | | |

# **Table S2: Baseline characteristics of the study population, by SNAC cohort and in the harmonized dataset stratified by age.**

| **<78 years** |  | | | | | | | | | |  |
| --- | --- | --- | --- | --- | --- | --- | --- | --- | --- | --- | --- |
|  | **SNAC-K**  **(n=1,700)** | | **SNAC-B**  **(n=543)** | | **SNAC-GÅS**  **(n=1,610)** | | **SNAC-N**  **(n=305)** | | **Harmonized dataset (n=4,157)** | |  |
| **Age, mean SD** | 65.3 | 4.8 | 65.9 | 4.8 | 65.1 | 4.8 | 66.2 | 4.8 | 65.4 | 4.8 |  |
| **Female, n %** | 976 | 57.4 | 299 | 55.1 | 825 | 51.2 | 149 | 48.8 | 2,249 | 54.1 |  |
| **Education, n %** |  |  |  |  |  |  |  |  |  |  |  |
| Primary school or below | 139 | 8.2 | 245 | 45.1 | 516 | 32.1 | 203 | 66.6 | 1,102 | 26.5 |  |
| High school | 780 | 45.9 | 209 | 38.5 | 725 | 45.0 | 79 | 25.9 | 1,793 | 43.1 |  |
| University of higher | 781 | 45.9 | 89 | 16.4 | 369 | 22.9 | 23 | 7.5 | 1,262 | 30.4 |  |
| **Gait speed (m/s), mean SD** | 1.2 | 0.3 | 1.3 | 0.3 | 1.4 | 0.2 | 1.0 | 0.3 | 1.3 | 0.3 |  |
| **MMSE, mean SD** | 29.1 | 1.6 | 28.1 | 2.5 | 27.5 | 2.5 | 29.0 | 2.2 | 28.3 | 2.3 |  |
| **Chronic diseases, mean SD** | 3.0 | 1.9 | 2.2 | 1.8 | 4.0 | 2.0 | 1.5 | 1.4 | 3.2 | 2.0 |  |
| **I-ADL, mean SD** | 0.1 | 0.5 | 0.4 | 0.8 | 0.3 | 0.8 | 0.3 | 1.1 | 0.2 | 0.7 |  |
| **P-ADL, mean SD** | 0.0 | 0.1 | 0.0 | 0.2 | 0.0 | 0.3 | 0.0 | 0.1 | 0.0 | 0.2 |  |
| **1-year mortality, n %** | 5 | 0.3 | 5 | 0.9 | 7 | 0.4 | 2 | 0.7 | 19 | 0.5 |  |
| **3-year mortality, n%** | 45 | 2.7 | 15 | 2.8 | 37 | 2.3 | 13 | 4.3 | 110 | 2.7 |  |
| **5-year mortality, n%** | 99 | 5.8 | 32 | 5.9 | 80 | 5.0 | 27 | 8.9 | 238 | 5.7 |  |
| **16-year mortality, n %** | 392 | 23.1 | 137 | 25.2 | 371 | 23.0 | 58 | 19.0 | 1,023 | 24.6 |  |
| **1-year unplanned admissions, n %** | 137 | 8.1 | 126 | 23.2 | 193 | 12.0 | 41 | 13.4 | 497 | 12.0 |  |
| **3-year unplanned admissions, n %** | 345 | 20.3 | 205 | 37.8 | 463 | 28.8 | 95 | 31.2 | 1,108 | 26.7 |  |
| **≥78 years** | |  | | | | | | | | | |
|  | | **SNAC-K**  **(n=1,396)** | | **SNAC-B**  **(n=685)** | | **SNAC-GÅS**  **(n=780)** | | **SNAC-N**  **(n=284)** | | **Harmonized dataset (n=3,145)** | |
| **Age, mean SD** | | 84.5 | 5.9 | 83.8 | 4.5 | 83.3 | 4.2 | 83.3 | 4.3 | 83.9 | 5.1 |
| **Female, n %** | | 1,007 | 72.1 | 408 | 59.6 | 467 | 59.9 | 162 | 57.0 | 2,044 | 65.0 |
| **Education, n %** | |  |  |  |  |  |  |  |  |  |  |
| Primary school or below | | 387 | 27.7 | 452 | 66.0 | 399 | 51.2 | 245 | 86.3 | 1,483 | 47.2 |
| High school | | 749 | 53.7 | 187 | 27.3 | 297 | 38.1 | 33 | 11.6 | 1,266 | 40.3 |
| University of higher | | 260 | 18.6 | 46 | 6.7 | 84 | 10.8 | 6 | 2.1 | 396 | 12.6 |
| **Gait speed (m/s), mean SD** | | 0.7 | 0.4 | 0.8 | 0.4 | 1.1 | 0.3 | 0.8 | 0.3 | 0.8 | 0.4 |
| **MMSE, mean SD** | | 26.2 | 5.8 | 24.8 | 5.3 | 25.7 | 3.2 | 27.4 | 3.4 | 25.9 | 5.0 |
| **Chronic diseases, mean SD** | | 5.2 | 2.5 | 3.2 | 2.3 | 6.0 | 2.3 | 2.6 | 1.9 | 4.8 | 2.6 |
| **I-ADL, mean SD** | | 1.5 | 2.3 | 1.6 | 2.2 | 0.9 | 1.6 | 1.5 | 2.3 | 1.4 | 2.1 |
| **P-ADL, mean SD** | | 0.3 | 1.0 | 0.4 | 1.1 | 0.2 | 0.7 | 0.1 | 0.5 | 0.3 | 1.0 |
| **1-year mortality, n %** | | 98 | 7.0 | 35 | 5.1 | 21 | 2.7 | 9 | 3.1 | 163 | 5.2 |
| **3-year mortality, n%** | | 318 | 22.8 | 137 | 20.0 | 100 | 12.8 | 58 | 20.4 | 613 | 19.5 |
| **5-year mortality, n%** | | 513 | 36.8 | 245 | 35.8 | 207 | 26.5 | 94 | 33.1 | 1,059 | 33.7 |
| **16-year mortality, n %** | | 1,159 | 83.0 | 608 | 88.8 | 682 | 87.4 | 207 | 72.9 | 2,714 | 86.3 |
| **1-year unplanned admissions, n %** | | 349 | 25.0 | 294 | 42.9 | 167 | 21.4 | 58 | 20.4 | 868 | 27.6 |
| **3-year unplanned admissions, n %** | | 713 | 51.1 | 449 | 65.6 | 406 | 52.1 | 148 | 52.1 | 1,716 | 54.6 |
| SNAC: Swedish National study on Aging and Care, K: Kungsholmen, B: Blekinge, GÅS: Skåne, N: Nordanstig, SD: standard deviation, MMSE: Mini-mental state examination, I-ADL: instrumental activities of daily living, P-ADL: Personal activities of daily living. | | | | | | | | | | | |

| **Table S3: Individual health indicator cut-off points across all SNAC sites and in the harmonized dataset.** | |  |
| --- | --- | --- |
| **SNAC-K** | |  |
| **Health indicator** | **Cut-off points** |  |
| P-ADL | 0, 1+ |  |
| I-ADL | 0-1, 2+ |  |
| Gait Speed | ≥1.5, (1.5-1], (1-0.4], <0.4 |  |
| Number of Chronic Diseases | 0, 1, 2-4, 5+ |  |
| MMSE | 30-29, 28-20, 19-0 |  |
| **SNAC-B** | | |
| **Health indicator** | | **Cut-off points** |
| P-ADL | | 0, 1+ |
| I-ADL | | 0-1, 2+ |
| Gait Speed | | ≥1.64, (1.64-1.33], (1.33-1.13], <1.13 |
| Number of Chronic Diseases | | 0-2, 3+ |
| MMSE | | 30, 29-25, 24-0 |
| **SNAC-GÅS** | |  |
| **Health indicator** | **Cut-off points** |  |
| P-ADL | 0-1, 2+ |  |
| I-ADL | 0, 1-2, 3+ |  |
| Gait Speed | ≥1.64, (1.64-1.33], (1.33-1.13], <1.13 |  |
| Number of Chronic Diseases | 0-2, 3-4, 5+ |  |
| MMSE | 30, 29, 28-20, 19-0 |  |
| **SNAC-N** | |  |
| **Health indicator** | **Cut-off points** |  |
| P-ADL | 0, 1+ |  |
| I-ADL | 0-1, 2+ |  |
| Gait Speed | ≥1.2, (1.2-0.8], <0.8 |  |
| Number of Chronic Diseases | 0, 1-2, 3+ |  |
| MMSE | 30, 29-28, 27-20, 19-0 |  |
| **Harmonized dataset** | | |
| **Health indicator** | | **Cut-off points** |
| P-ADL | | 0, 1+ |
| I-ADL | | 0-1, 2+ |
| Gait Speed | | ≥1.64, (1.64-1.1], <1.1 |
| Number of Chronic Diseases | | 0-1, 2-3, 4+ |
| MMSE | | 30-29, 28-20, 19-0 |

| **Table S4: Most frequent health indicator categories (frequency 50% and above) by HAT score in SNAC-K.** | | | | | | |
| --- | --- | --- | --- | --- | --- | --- |
| HAT Score | P-ADL | I-ADL | Gait Speed | Number of Chronic Diseases | MMSE | Count |
| 9.5-10 | 0 | 0-1 | ≥1.5 | 1 | 29-30 | 165 |
| 9.0-9.4 | 0 | 0-1 | ≥1.5 | 2-4 | 29-30 | 464 |
| 8.0-8.9 | 0 | 0-1 | 1.0-1.5 | 2-4 | 29-30 | 1162 |
| 7.0-7.9 | 0 | 0-1 | 1.0-1.5 | 5+ | 20-28 | 105 |
| 6.0-6.9 | 0 | 0-1 | 0.4-1.0 | 5+ | 29-30 | 483 |
| 5.0-5.9 | 0 | 0-1 | <0.4 | 5+ | 30-20* | 248 |
| 4.0-4.9 | 0 | 2+ | 0.4-1.0 | 5+ | 29-30 | 37 |
| 3.0-3.9 | 0 | 2+ | 0.4-1.0 | 5+ | 20-28 | 111 |
| 2.0-2.9 | 0 | 2+ | <0.4 | 5+ | 20-28 | 136 |
| 1.0-1.9 | 1+ | 2+ | <0.4 | 5+ | 20-28 | 111 |
| 0.0-0.9 | 1+ | 2+ | <0.4 | 5+ | ≤19 | 86 |
| * No category was above 50%, both categories 30-29 and 28-20 were the highest two | | | | | | |

| **Table S5: Most frequent health indicator categories (frequency 50% and above) by HAT score in SNAC-B.** | | | | | | |
| --- | --- | --- | --- | --- | --- | --- |
| HAT Score | P-ADL | I-ADL | Gait Speed | Number of Chronic Diseases | MMSE | Count |
| 9.5-10 | 0 | 0-1 | All below 50%, similar ratios | 0-2 | 25-29 | 916 |
| 9.0-9.4 | 0 | 0-1 | <1.13 | 3+ | ≤24 | 76 |
| 8.0-8.9 | 1+ | 0-1 | <1.13 | 3+ | 25-29 | 4 |
| 7.0-7.9 | 0/1+* | 0-1/2+* | 1.33-1.64/1.13-1.33* | 3+ | 30/≤24* | 2 |
| 6.0-6.9 | 0/1+* | 0-1/2+* | <1.13 | 0-2 | 25-29/≤24* | 8 |
| 5.0-5.9 | 0 | 2+ | 1.33-1.13 | 3+ | 25-29 | 5 |
| 4.0-4.9 | 0 | 2+ | <1.13 | 3+ | 25-29 | 59 |
| 3.0-3.9 | 0 | 2+ | <1.13 | 3+ | ≤24 | 48 |
| 2.0-2.9 | 1+ | 2+ | <1.13 | 0-2 | 25-29 | 10 |
| 1.0-1.9 | 1+ | 2+ | <1.13 | 3+ | 25-29 | 20 |
| 0.0-0.9 | 1+ | 2+ | <1.13 | 3+ | ≤24 | 80 |
| * No category was above 50%, highest categories shown | | | | | | |
| **Table S6: Most frequent health indicator categories (frequency 50% and above) by HAT score in SNAC-GÅS.** | | | | | | |
| HAT Score | P-ADL | I-ADL | Gait Speed | Number of Chronic Diseases | MMSE | Count |
| 9.5-10 | 0-1 | 0 | 1.33-1.64 | 0-2 | 29* | 552 |
| 9.0-9.4 | 0-1 | 0 | 1.33-1.64 | 3-4 | 20-28 | 405 |
| 8.0-8.9 | 0-1 | 0 | 1.13-1.33 | 5+ | 20-28 | 553 |
| 7.0-7.9 | 0-1 | 0 | 1.13-1.33 | 5+ | 20-28 | 328 |
| 6.0-6.9 | 0-1 | 0 | <1.13 | 5+ | 20-28 | 333 |
| 5.0-5.9 | 0-1 | 1-2 | <1.13 | 5+ | 20-28 | 106 |
| 4.0-4.9 | 0-1 | 3+ | <1.13 | 5+ | 20-28 | 88 |
| 3.0-3.9 | 0-1 | 3+ | <1.13 | 5+ | ≤19 | 7 |
| 2.0-2.9 | NA | NA | NA | NA | NA | 0 |
| 1.0-1.9 | 2+ | 1-2 | <1.13 | 5+ | 20-28 | 6 |
| 0.0-0.9 | 2+ | 3+ | <1.13 | 5+ | 20-28 | 39 |
| * No category was above 50%, category 29 at 40% | | | | | | |

| **Table S7: Most frequent health indicator categories (frequency 50% and above) by HAT score in SNAC-N.** | | | | | | |
| --- | --- | --- | --- | --- | --- | --- |
| HAT Score | P-ADL | I-ADL | Gait Speed | Number of Chronic Diseases | MMSE | Count |
| 9.5-10 | 0 | 0-1 | ≥1.2 | 0 | 30 | 74 |
| 9.0-9.4 | 0 | 0-1 | ≥1.2 | 1-2 | 28-29 | 46 |
| 8.0-8.9 | 0 | 0-1 | 0.8-1.2 | 1-2 | 28-29 | 258 |
| 7.0-7.9 | 0 | 0-1 | 0.8-1.2 | 1-2 | 20-27 | 31 |
| 6.0-6.9 | 0 | 0-1 | <0.8 | 3+ | 28-29 | 65 |
| 5.0-5.9 | 0 | 0-1/2+* | <0.8 | 3+ | 20-27 | 16 |
| 4.0-4.9 | 0 | 0-1/2+* | <0.8 | 3+ | ≤19 | 12 |
| 3.0-3.9 | 0 | 2+ | <0.8 | 3+ | 20-28 | 61 |
| 2.0-2.9 | 0 | 2+ | <0.8 | 0/1-2/3+^#^ | ≤19 | 6 |
| 1.0-1.9 | 1+ | 2+ | <0.8 | 3+ | 20-28 | 9 |
| 0.0-0.9 | 1+ | 2+ | <0.8 | 3+ | ≤19 | 12 |
| * No category was above 50%, both categories scored 50%  # No category was above 50%, all categories scored 33% | | | | | | |
| **Table S8: Most frequent health indicator categories (frequency 50% and above) by HAT score in the harmonized dataset.** | | | | | | |
| HAT Score | P-ADL | I-ADL | Gait Speed | Number of Chronic Diseases | MMSE | Count |
| 9.5-10 | 0 | 0-1 | 1.64-1.1 | 2-3 | 30-29 | 1497 |
| 9.0-9.4 | 0 | 0-1 | 1.64-1.1 | 4+^a^ | 28-20 | 1592 |
| 8.0-8.9 | 0 | 0-1 | <1.1 | 4+ | 30-29 | 2110 |
| 7.0-7.9 | 0 | 0-1 | <1.1 | 4+ | 28-20 | 947 |
| 6.0-6.9 | 1+ | 0-1 | <1.1 | 4+ | 19-0^b^ | 64 |
| 5.0-5.9 | 0 | 2+ | 1.64-1.1 | 4+ | 28-20 | 82 |
| 4.0-4.9 | 0 | 2+ | <1.1 | 4+ | 28-20 | 280 |
| 3.0-3.9 | 0 | 2+ | <1.1 | 4+ | 28-20 | 266 |
| 2.0-2.9 | 1+ | 2+ | <1.1 | 4+ | 19-0^c^ | 151 |
| 1.0-1.9 | 1+ | 2+ | <1.1 | 4+ | 28-20 | 147 |
| 0.0-0.9 | 1+ | 2+ | <1.1 | 4+ | 19-0 | 167 |
| ^a^ All categories below 50%. Highest category reported (4+) at 48%  ^b^ All categories below 50%. Highest category reported (19-0) at 41%  ^c^ All categories below 50%. Highest category reported (19-0) at 46% | | | | | | |

# **Figure S1: Test Information Function across all SNAC sites and in the harmonized dataset.**


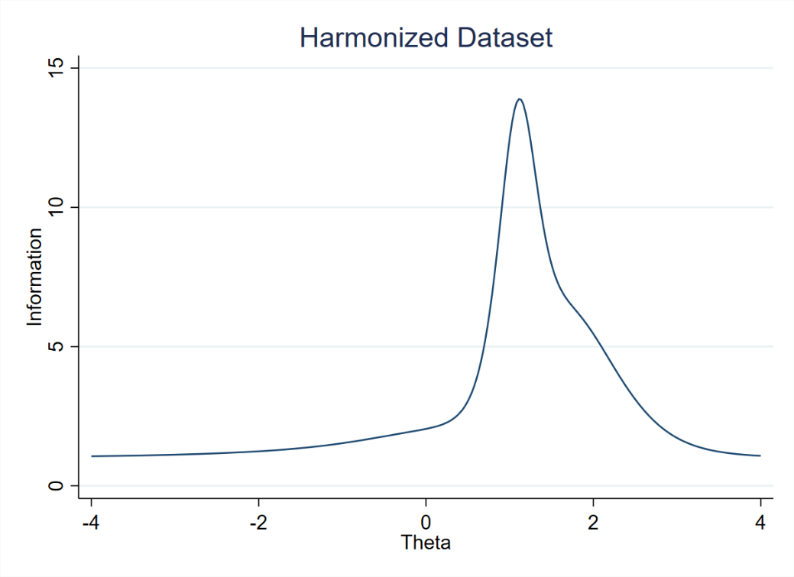


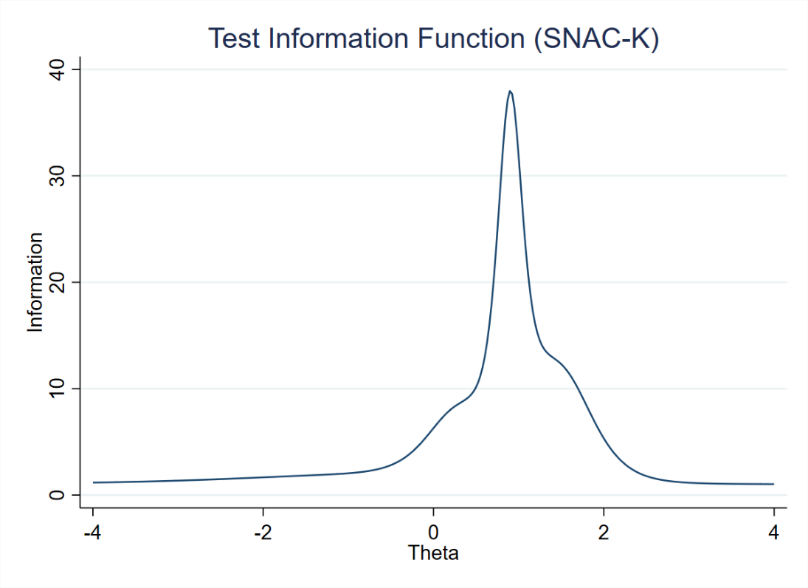


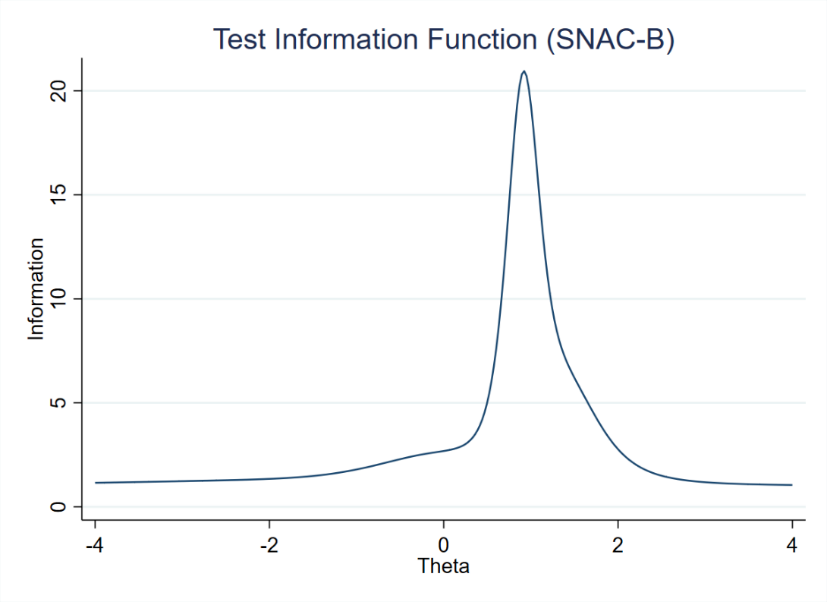


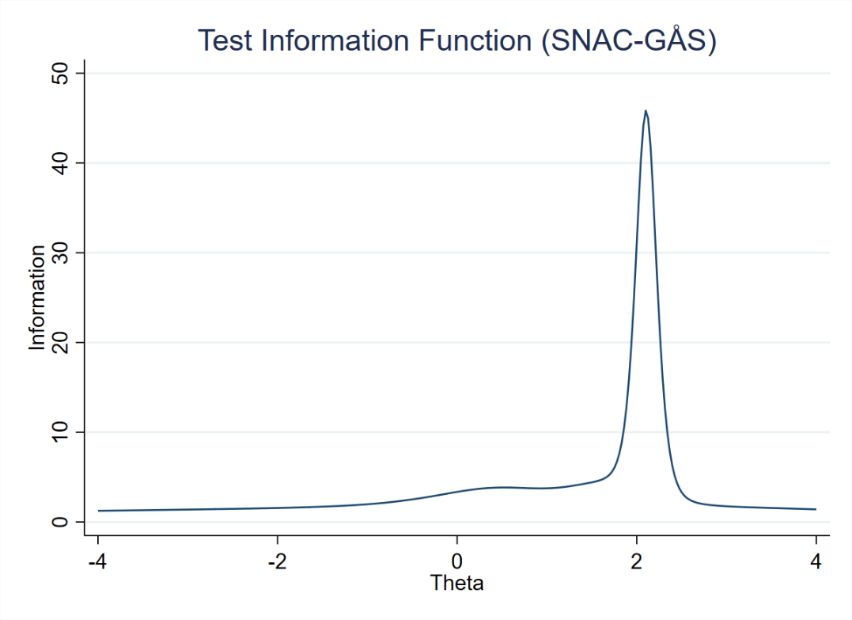


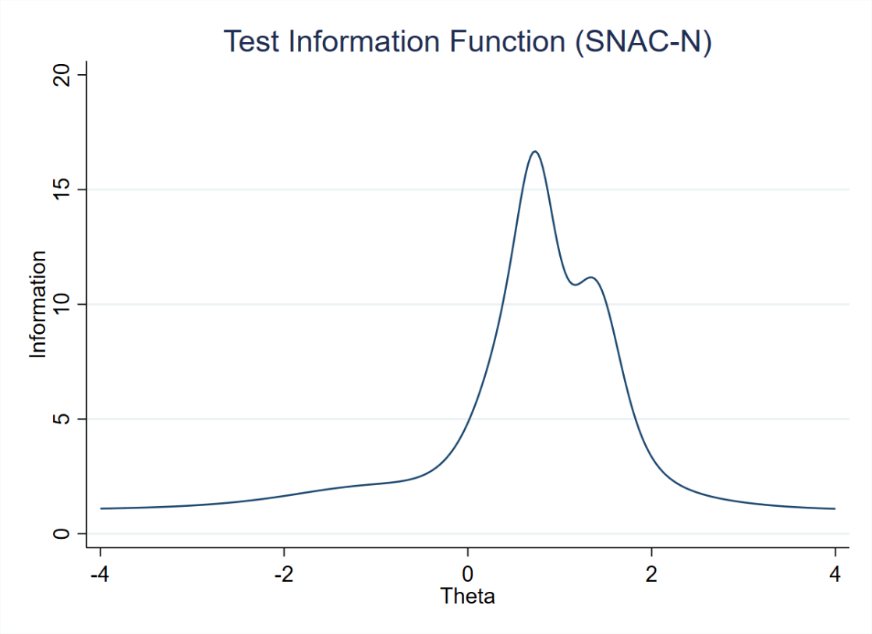


# **Figure S2: Individual and meta-analyzed predictive capacity of the HAT across all SNAC sites except for SNAC-B.**


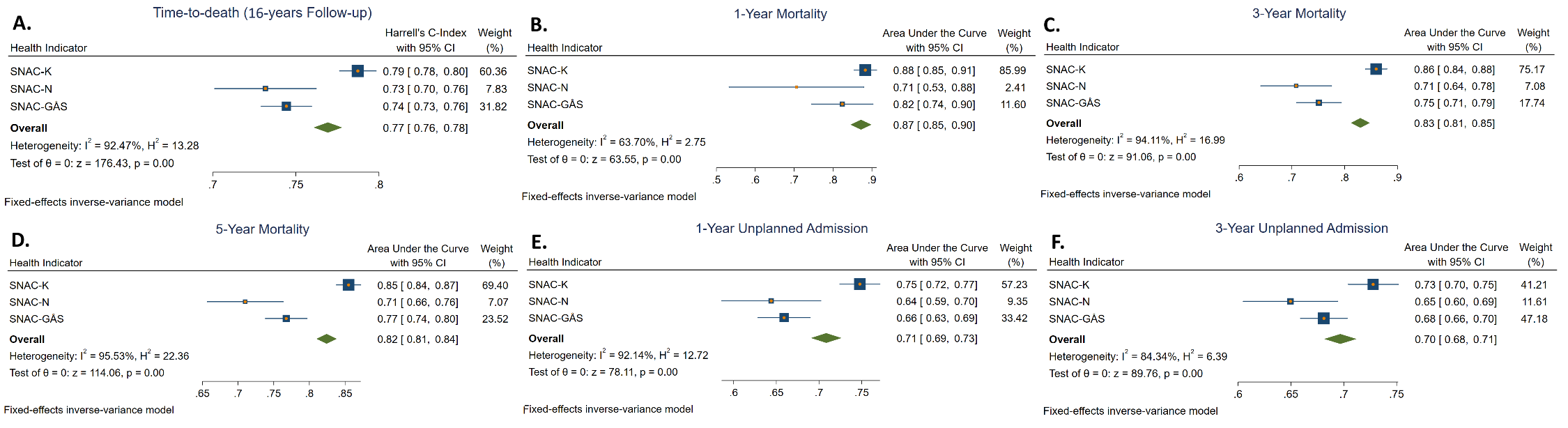


# **Table S9: Predictive capacity of the HAT across SNAC sites and in the harmonized dataset, overall and stratified by sex and age.**

|  | **SNAC-K** | **SNAC-B** | **SNAC- GÅS** | **SNAC-N** | **Harmonized dataset** |
| --- | --- | --- | --- | --- | --- |
|  | **PE (95% CI)** | **PE (95% CI)** | **PE (95% CI)** | **PE (95% CI)** | **PE (95% CI)** |
| Time-to-death (16-years follow-up)**^a^** | | | | | |
| Overall | 0.79  (0.78, 0.80) | 0.72  (0.71, 0.74) | 0.74  (0.73, 0.76) | 0.73  (0.70, 0.76) | 0.75  (0.74,0.75) |
| Males | 0.77  (0.76, 0.79) | 0.64  (0.61, 0.68) | 0.71  (0.69, 0.73) | 0.76  (0.71, 0.80) | 0.73  (0.72, 0.74) |
| Females | 0.80  (0.79, 0.81) | 0.66  (0.64, 0.69) | 0.72  (0.70, 0.74) | 0.72  (0.67, 0.76) | 0.76  (0.75, 0.77) |
| <78 years | 0.69  (0.66, 0.72) | 0.52  (0.47, 0.58) | 0.64  (0.61, 0.67) | 0.68  (0.60, 0.76) | 0.64  (0.62, 0.66) |
| ≥78 years | 0.71  (0.69, 0.72) | 0.65  (0.63, 0.67) | 0.63  (0.61, 0.65) | 0.63  (0.59, 0.67) | 0.67  (0.66, 0.68) |
| 1-year mortality**^b^** | | | | | |
| Overall | 0.88  (0.85, 0.91) | 0.81  (0.73, 0.88) | 0.82  (0.75, 0.90) | 0.71  (0.53, 0.88) | 0.84  (0.81, 0.87) |
| Males | 0.91  (0.87, 0.95) | 0.79  (0.69, 0.88) | 0.90  (0.84, 0.96) | 0.82  (0.71, 0.92) | 0.85  (0.81, 0.89) |
| Females | 0.88  (0.84, 0.91) | 0.88  (0.81, 0.95) | 0.70  (0.52, 0.88) | 0.61  (0.20, 1.00) | 0.85  (0.81, 0.89) |
| <78 years | 0.71  (0.41, 1.00) | 0.68  (0.42, 0.94) | 0.79  (0.56, 1.00) | 0.70  (0.25, 1.00) | 0.74  (0.60, 0.87) |
| ≥78 years | 0.80  (0.76, 0.84) | 0.76  (0.68, 0.85) | 0.70  (0.58, 0.82) | 0.63  (0.42, 0.85) | 0.77  (0.73, 0.80) |
| 3-year mortality**^b^** | | | | | |
| Overall | 0.86  (0.84, 0.88) | 0.81  (0.77, 0.85) | 0.75  (0.71, 0.79) | 0.71  (0.64, 0.78) | 0.81  (0.80, 0.83) |
| Males | 0.85  (0.82, 0.89) | 0.79  (0.73, 0.84) | 0.80  (0.75, 0.85) | 0.77  (0.68, 0.86) | 0.80  (0.78, 0.83) |
| Females | 0.87  (0.84, 0.89) | 0.84  (0.80, 0.89) | 0.76  (0.70, 0.83) | 0.65  (0.55, 0.74) | 0.83  (0.81, 0.85) |
| <78 years | 0.74  (0.65, 0.83) | 0.74  (0.60, 0.88) | 0.62  (0.52, 0.72) | 0.57  (0.38, 0.77) | 0.69  (0.63, 0.74) |
| ≥78 years | 0.79  (0.76, 0.82) | 0.74  (0.69, 0.79) | 0.67  (0.62, 0.73) | 0.62  (0.54, 0.71) | 0.74  (0.72, 0.76) |
| 5-year mortality**^b^** | | | | | |
| Overall | 0.86  (0.84, 0.87) | 0.80  (0.77, 0.83) | 0.77  (0.74, 0.80) | 0.71  (0.66, 0.76) | 0.80  (0.79, 0.82) |
| Males | 0.85  (0.82, 0.88) | 0.80  (0.76, 0.85) | 0.79  (0.75, 0.82) | 0.77  (0.70, 0.84) | 0.79  (0.77, 0.81) |
| Females | 0.87  (0.85, 0.89) | 0.81  (0.78, 0.85) | 0.77  (0.72, 0.81) | 0.67  (0.59, 0.74) | 0.82  (0.81, 0.84) |
| <78 years | 0.73  (0.68, 0.79) | 0.66  (0.56, 0.76) | 0.66  (0.60, 0.73) | 0.60  (0.47, 0.73) | 0.67  (0.63, 0.71) |
| ≥78 years | 0.80  (0.77, 0.82) | 0.75  (0.71, 0.78) | 0.66  (0.62, 0.71) | 0.63  (0.56, 0.70) | 0.74  (0.72, 0.75) |
| 1-year unplanned hospital admissions**^b^** | | | | | |
| Overall | 0.75  (0.72, 0.77) | 0.66  (0.63, 0.69) | 0.66  (0.63, 0.69) | 0.64  (0.59, 0.70) | 0.69  (0.67, 0.70) |
| Males | 0.75  (0.71, 0.79) | 0.66  (0.61, 0.71) | 0.64  (0.60, 0.69) | 0.74  (0.66, 0.81) | 0.68  (0.65, 0.70) |
| Females | 0.75  (0.72, 0.78) | 0.66  (0.61, 0.70) | 0.69  (0.65, 0.74) | 0.54  (0.45, 0.63) | 0.70  (0.68, 0.72) |
| <78 years | 0.67  (0.62, 0.72) | 0.54  (0.49, 0.59) | 0.64  (0.60, 0.68) | 0.69  (0.60, 0.77) | 0.62  (0.59, 0.65) |
| ≥78 years | 0.68  (0.65, 0.71) | 0.65  (0.61, 0.69) | 0.62  (0.57, 0.67) | 0.58  (0.49, 0.66) | 0.64  (0.62, 0.66) |
| 3-year unplanned hospital admissions**^b^** | | | | | |
| Overall | 0.73  (0.71, 0.75) | 0.66  (0.64, 0.69) | 0.68  (0.66, 0.70) | 0.65  (0.61, 0.69) | 0.69  (0.68, 0.70) |
| Males | 0.73  (0.70, 0.76) | 0.65  (0.61, 0.70) | 0.66  (0.62, 0.69) | 0.67  (0.60, 0.73) | 0.68  (0.66, 0.70) |
| Females | 0.73  (0.71, 0.75) | 0.67  (0.63, 0.71) | 0.71  (0.68, 0.74) | 0.63  (0.57, 0.69) | 0.70  (0.68, 0.72) |
| <78 years | 0.64  (0.61, 0.67) | 0.52  (0.48, 0.56) | 0.63  (0.60, 0.66) | 0.63  (0.56, 0.70) | 0.61  (0.59, 0.63) |
| ≥78 years | 0.64  (0.61, 0.67) | 0.65  (0.61, 0.70) | 0.63  (0.59, 0.67) | 0.57  (0.50, 0.64) | 0.63  (0.61, 0.65) |
| HAT: Health Assessment Tool, PE: Point Estimate, CI: Confidence Interval  ^a^ Harrell’s C-statistics  ^b^ Area Under the Curve (AUC) of the Receiver Operating Characteristic curve (ROC) | | | | | |

# **Figure S3: HAT-based geriatric charts and risk of 5-years mortality stratified by sex across all SNAC sites and in the harmonized dataset.**


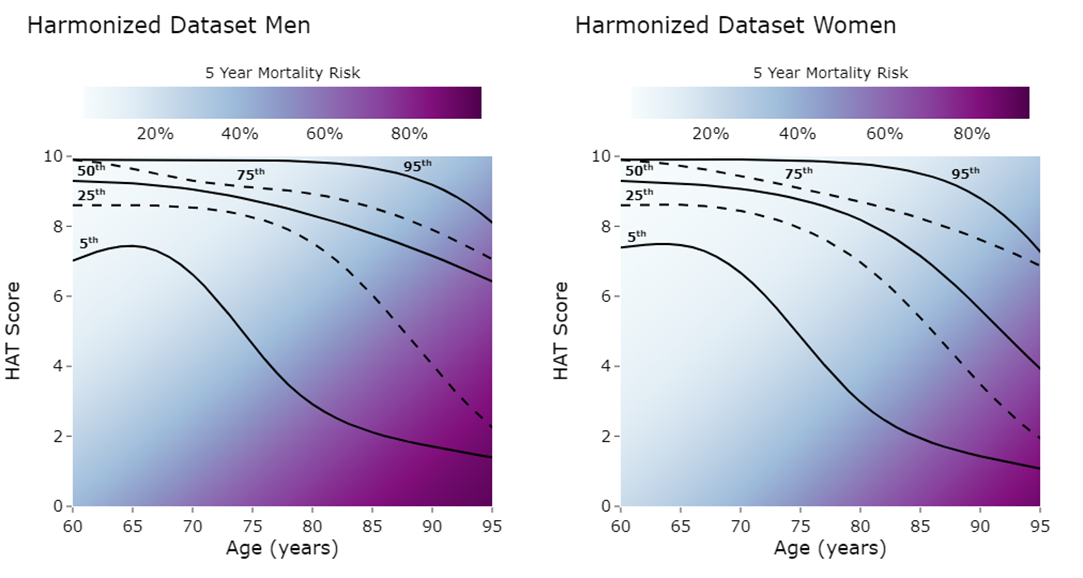


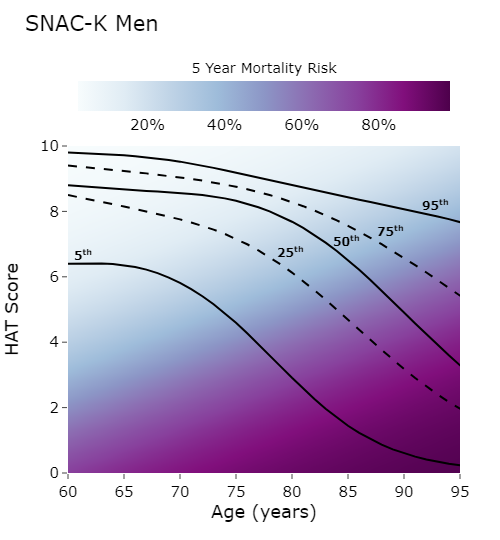

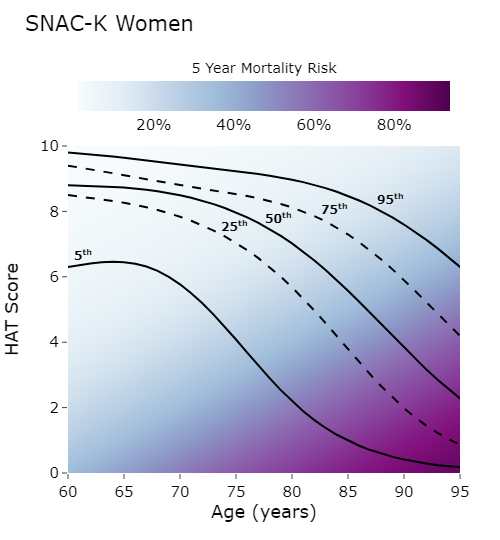

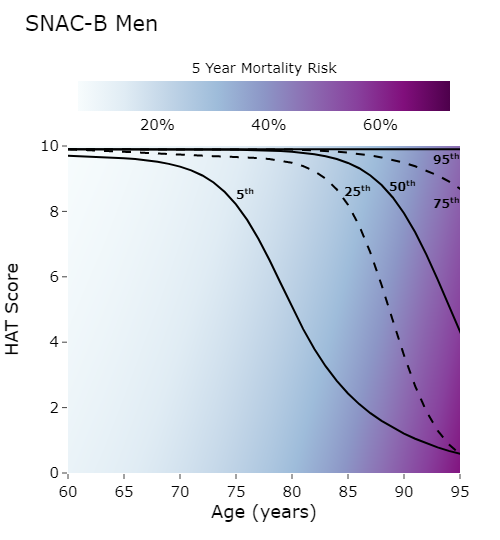

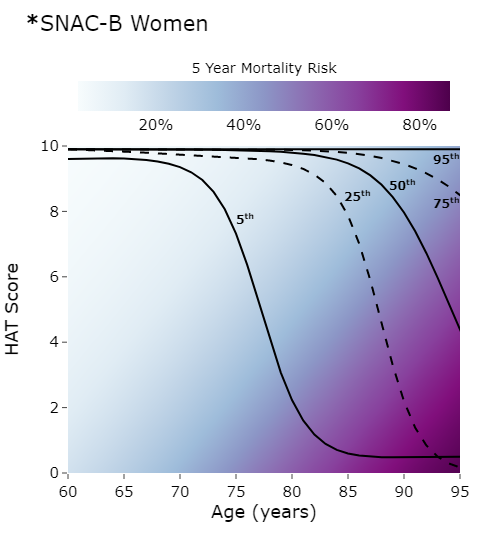


******* *Slight overlap in percentile curves at higher ages due to modelling issues caused by smaller sample sizes within logistic quantile regression.*


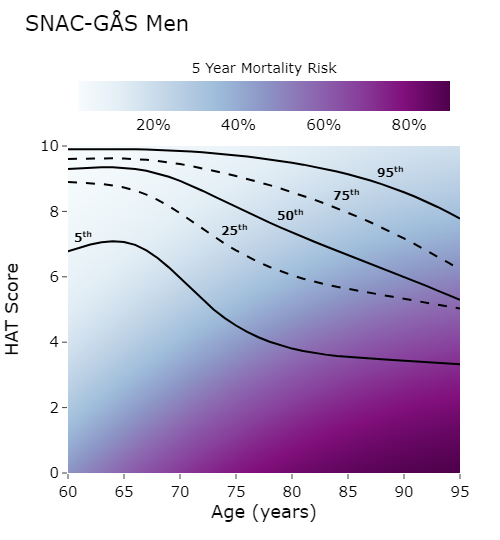

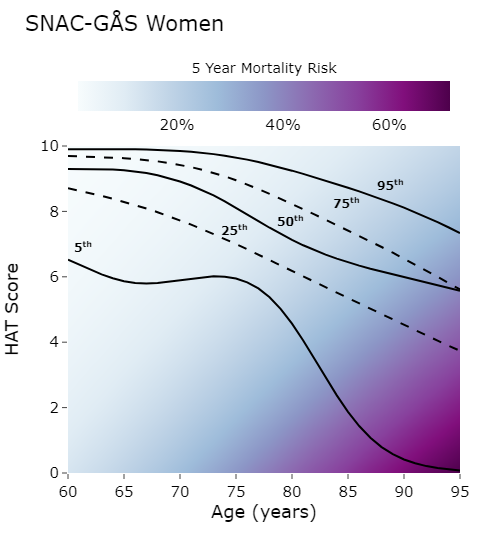

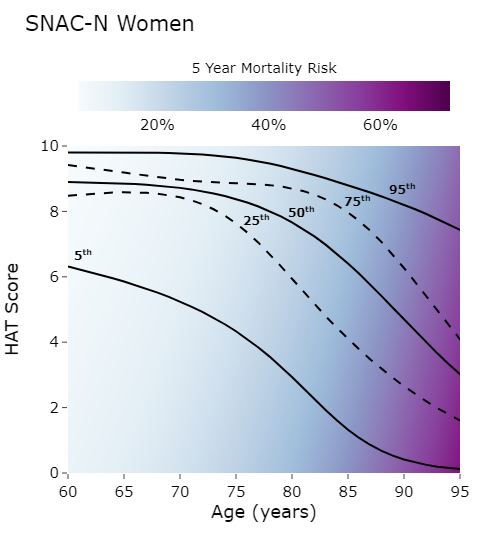

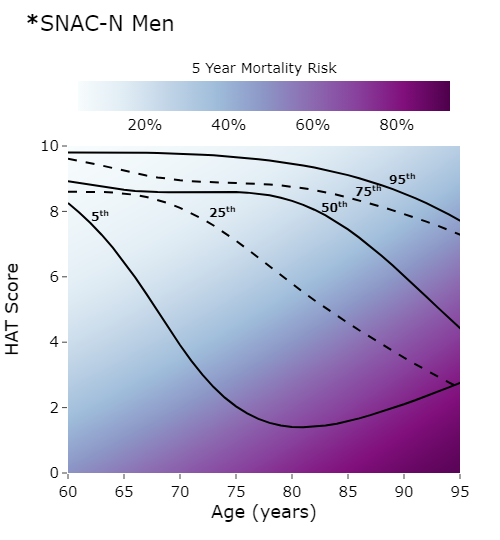


******* *Slight overlap in percentile curves at higher ages due to modelling issues caused by smaller sample sizes within logistic quantile regression.*

# **Figure S4: Reliability plots for different SNAC sites and the harmonized dataset, divided into 10 equal sized groups (percentiles) with 5-year mortality as the outcome.**


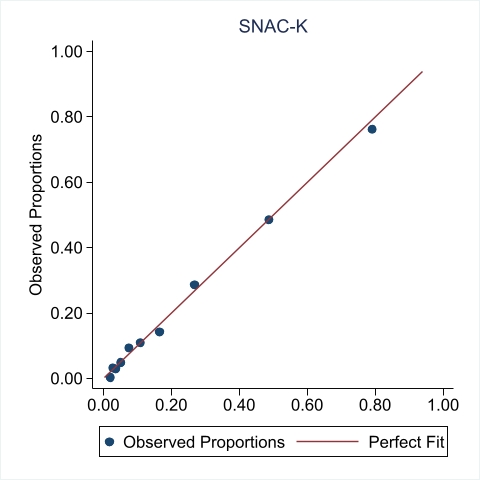

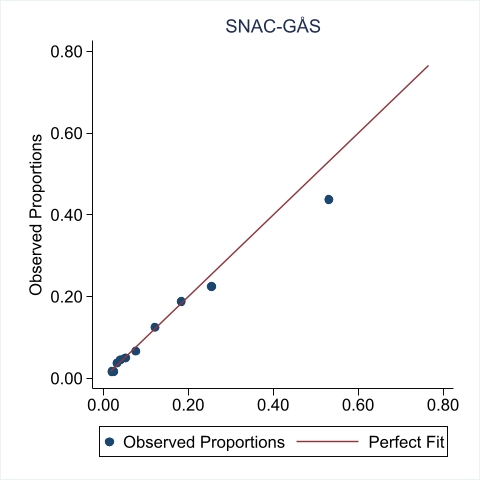

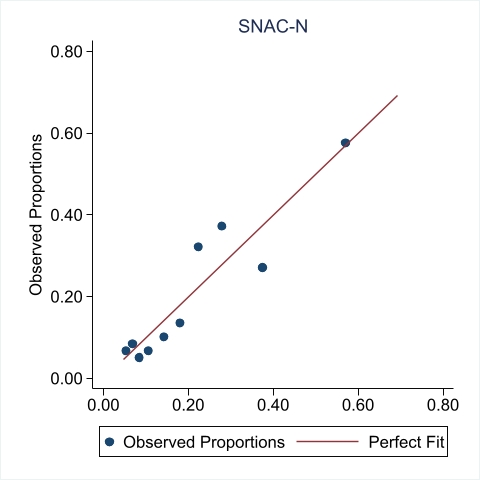

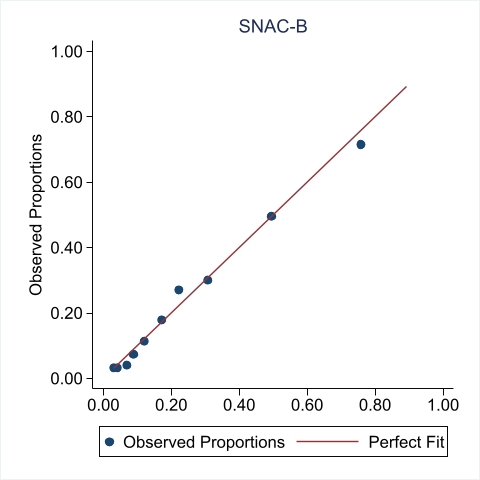

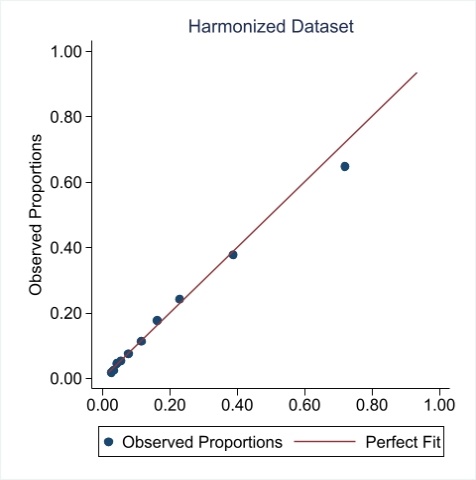

Supplement: Supplementary file 1 — Additional file 1: Document S1, Tables S1–S9, Figures S1–S4. Document S1 Calculation of HAT scores, Table S1 Baseline characteristics of the study population across SNAC sites stratified by sex, Table S2 Baseline characteristics of the study population across SNAC sites stratified by age, Table S3 Individual health indicator cut-off points, Table S4 Most frequent health indicator categories by HAT score in SNAC-K, Table S5 Most frequent health indicator categories by HAT score in SNAC-B, Table S6 Most frequent health indicator categories by HAT score in SNAC-GÅS, Table S7 Most frequent health indicator categories by HAT score in SNAC-N, Table S8 Most frequent health indicator categories by HAT score in harmonized dataset, Figure S1 Test information function, Figure S2 Individual and meta-analyzed predictive capacity of the HAT across all SNAC sites except for SNAC-B, Table S9 Predictive capacity of the HAT, Figure S3 HAT-based geriatric charts and risk of 5-year mortality, Figure S4 Reliability plots. [file 12916_2024_3454_MOESM1_ESM.docx]
